# Impact of BECLIN1 haploinsufficiency on goblet cell function and susceptibility to colitis

**DOI:** 10.1038/s41419-026-08984-8

**Published:** 2026-06-17

**Authors:** Juliani Juliani, Sharon Tran, Tiffany J. Harris, Sarah L. Ellis, Aysha H. Al-Ani, Yashodha Wijebandara, Komal M. Patel, Samuel N. Young, Marco Evangelista, David Baloyan, Camilla M. Reehorst, Rebecca Nightingale, Laura J. Jenkins, Peter De Cruz, Kinga Duszyc, Benjamin T. Kile, Alpha S. Yap, John M. Mariadason, Britt Christensen, André L. Samson, James M. Murphy, Walter D. Fairlie, Erinna F. Lee

**Affiliations:** 1 Department of Biochemistry and Chemistry, School of Agriculture, Biomedicine and Environment, La Trobe University, Bundoora, VIC Australia; 2 La Trobe Institute for Molecular Science, La Trobe University, Bundoora, VIC Australia; 3 Olivia Newton-John Cancer Research Institute, Heidelberg, VIC Australia; 4 School of Cancer Medicine, La Trobe University, Bundoora, VIC Australia; 5 Walter and Eliza Hall Institute of Medical Research, Parkville, VIC Australia; 6 University of Melbourne, Parkville, VIC Australia; 7 Royal Melbourne Hospital, Parkville, VIC Australia; 8 Department of Gastroenterology, Austin Health, Melbourne, VIC Australia; 9 Department of Medicine, Austin Health, The University of Melbourne, Melbourne, VIC Australia; 10 Institute for Molecular Bioscience, The University of Queensland, St. Lucia, Brisbane, QLD Australia; 11 Garvan Institute of Medical Research, Darlinghurst, NSW Australia; 12 Drug Discovery Biology, Monash Institute of Pharmaceutical Sciences, Monash University, Parkville, VIC Australia

**Keywords:** Mechanisms of disease, Crohn's disease

## Abstract

BECLIN1 is a central regulator of autophagy and endocytic trafficking essential for epithelial homoeostasis. While complete intestinal epithelial loss of BECLIN1 causes fatal enteritis originating in the small intestine, the consequences of its partial loss in the gut remain unclear. Given that BECLIN1 expression can vary in human disease, we investigated whether reduced BECLIN1 is sufficient to impair gut barrier function. Heterozygous *Becn1* deletion (*Becn1*^*IEC+/−*^) in the mouse intestinal epithelium caused subtle but significant defects. These included shortened small intestines and altered epithelial architecture, despite preservation of basal autophagy, implicating trafficking-related functions. Supporting this conclusion, *Becn1*^*IEC+/−*^ small intestinal epithelial cells showed modest increases in RAB5^+ve^ vesicles, redistribution of E-CADHERIN and F-actin along lateral membranes and altered apico-basal cell morphology. Given the absence of overt small intestinal epithelial disruption or inflammation, as seen with complete loss of BECLIN1, we next addressed whether BECLIN1 insufficiency manifests a phenotype under stress or in other gut regions. Indeed, in the colon, *Becn1*^*IEC+/−*^ mice exhibited reduced colonic crypt length, baseline goblet cell loss and reduced mucin production, particularly in mature goblet cells, indicating vulnerability of the mucus barrier. When challenged with dextran sulfate sodium (DSS), *Becn1*^*IEC+/−*^ mice exhibited greater weight loss, higher disease activity, more severe histological colitis, and disproportionate loss of neutral mucins, with inflammation confined to the mucosa. Together, these findings show that BECLIN1 insufficiency does not trigger spontaneous inflammation but destabilises epithelial organisation and barrier defence, thereby sensitising the gut to inflammatory challenge and further positioning BECLIN1 as a threshold-dependent determinant of intestinal resilience.

## Introduction

Genome-wide association studies have identified polymorphisms in autophagy regulators, such as *ATG16L1* and *IRGM*, as strongly associated with inflammatory bowel disease (IBD) [1–7]. These findings highlight the importance of autophagy in intestinal homoeostasis and implicate its disruption in IBD pathogenesis. Functional studies in mouse and cell-based models have revealed both epithelial-intrinsic roles, including maintenance of barrier integrity and cell survival [2, 8–16], and immune-mediated extrinsic roles, such as regulating inflammatory responses and microbial balance [17–21].

Recent studies have shown that the prototypical autophagy regulator BECLIN1 is critical for intestinal homoeostasis. Constitutive activation of BECLIN1-dependent autophagy alleviates goblet cell ER stress, thickening the mucus barrier and protecting against chemical- and infection-driven inflammation [11]. Beyond autophagy, BECLIN1 regulates endocytic trafficking, maintaining barrier integrity via correct junctional protein localisation, cytoskeletal organisation, and epithelial remodelling [22–24]. Complete intestinal epithelial deletion of BECLIN1 is rapidly fatal due to severe enterocolitis with extensive epithelial cell loss, impaired specialised IEC functions, inflammation, and barrier breakdown [23].

BECLIN1 undergoes extensive post-translational modifications that fine-tune its activity and interactions [25–28]. In diseases, however, it is reduced BECLIN1 expression rather than mutation that contributes to pathogenesis [29–31], with changes arising from altered protein interactions, cleavage, epigenetic regulation, or monoallelic deletion [32–35]. Despite this, the consequences of partial BECLIN1 loss in the intestinal epithelium remain poorly understood. Here, we demonstrate that partial loss of BECLIN1 impairs goblet cell function and increases susceptibility to inflammation in the colon. By dissecting its roles in endocytic trafficking, cytoskeletal organisation and mucin secretion, we provide new insights into how BECLIN1 insufficiency destabilises epithelial integrity and compromises barrier defence.

## Materials and methods

### Animal experiments

All mouse strains used in this study were bred on the C57BL/6 J background. Becn1^tm1b(KOMP)Wtsi^ mice were purchased from the European Conditional Mouse Mutagenesis Program (EUCOMM). *Becn1*^*fl/fl*^ mice were generated by breeding Becn1^tm1b(KOMP)Wtsi^ mice onto CAG-FLPe mice. *Becn1*^*+/+*^*;*, *Becn1*^*fl/+*^*;*, and *Becn1*^*fl/fl*^;*Vil1-CreERT2*^*Cre/+*^ mice were then subsequently generated by breeding *Becn1*^*+/+*^, *Becn1*^*fl/+*^ and *Becn1*^*fl/fl*^ mice to the Vil1-CreERT2 mice [36].

Mice were housed at the La Trobe Animal Research and Teaching Facility (LARTF, La Trobe University, VIC, Australia) under Specific Pathogen Free (SPF) conditions. All experiments performed were approved by the La Trobe University animal ethics committees (approvals AEC18024, AEC18036) in accordance with the Australian code for the care and use of animals for scientific purposes. All research with these mice has complied with all relevant ethical regulations for animal use. To induce gene deletion, male and female mice aged between 6 and 17 weeks (unless otherwise specified), were selected indiscriminately and intraperitoneally injected with 4 mg tamoxifen (T5648, Sigma-Aldrich, St. Louis, Missouri, USA) in sunflower seed oil (25007, Sigma-Aldrich, St. Louis, Missouri, USA), delivered as one 200 μl injection per day of a 10 mg/ml stock, over two consecutive days. The study was not powered for sex-specific analyses. Mice were humanely euthanised by CO_2_ asphyxiation.

Animals were allocated to experimental groups based on genotype and no formal randomisation was applied. Mice were otherwise selected indiscriminately with respect to sex, weight, and cage origin, and all outcome analyses were performed blinded to genotype to minimise bias.

### Dextran Sulphate Sodium (DSS) treatment

Following Tamoxifen treatment to induce gene deletion, 7–14 weeks old mice were administered DSS (2% (w/v), MP Biomedicals, Irvine, California, USA) in autoclaved drinking water provided *ad libitum* for 5 days. Control groups received autoclaved drinking water without DSS for the same duration. During the experimental period, mice were monitored daily and weighed. The average daily intake of 2% (w/v) DSS water was calculated by measuring the weight difference of drinking bottles at the start and end of the experiment and dividing by the number of treatment days. The disease activity index (DAI) was assessed using a cumulative scoring system based on parameters outlined in Table 1, including weight loss, stool consistency and the presence of hematochezia (presence of blood in stool). At the experimental endpoint, mice were humanely euthanised using CO_2_ asphyxiation. The small and large intestine were harvested, and their lengths were measured prior to rolling and fixation in 10% (v/v) neutral buffered formalin for 48 h.

**Table 1:** Scoring system for the evaluation of colitis severity in mice based on weight loss, stool consistency and haematochezia parameters.

Score	Weight loss (%)	Stool consistency	Haematochezia
0	Normal	Normal	No blood
1	<5	-	Visible blood in rectum
2	6–10	Pasty, semiformed	Visible blood on fur
3	11–20	-	-
4	>20	Liquid, sticky or unable to defecate after 5 min	-

### Intestinal organoid culture

Organoids were established by culturing crypt-enriched fractions from the duodenum of 8–12 weeks old untreated mice as described previously [23]. To induce *Becn1* deletion, organoids were seeded into media containing 200 nM 4-hydroxytamoxifen (4-HT) (H7904, Sigma-Aldrich, St. Louis, Missouri, USA) for three days and then maintained as per normal. For organoid re-passaging experiments, 4-HT-treated organoids were passaged at day 7 and maintained as above.

### Intestinal epithelial cell isolation

The mouse small intestine was opened longitudinally, rinsed with Dulbecco’s Phosphate Buffered Saline (dPBS), and incubated in dPBS + 15 mM EDTA for 15 min at 37 °C with agitation. Dissociation of IECs was achieved by vortexing. The cell suspension was then washed once with ice-cold dPBS, and the cell pellet snap-frozen on dry ice and stored at −80 °C until use.

### Histology, immunohistochemistry and organoid whole-mount immunofluorescence

Preparation of intestinal “Swiss-rolls”, haematoxylin and eosin (H&E), Periodic acid-Schiff, Alcian blue (PAS-AB) and Ki-67 staining were all performed as described previously [23]. All slides were scanned using the Aperio AT2 (Leica, Wetzlar, Germany) and images captured and analysed using Aperio ImageScope v12.4.2.5010 and Fiji ImageJ. Whole-mount staining of intestinal organoids was performed as previously described [22]. Detailed image acquisition and quantification procedures are described in the Supplementary information.

### Immunoblotting of mouse tissues

Preparation of mouse cell lysates and immunoblotting was performed as described previously [23]. Primary antibodies were: Beclin1, 1:500 (3495, Cell Signalling Technology, Danvers, Massachusetts, USA); p62/SQSTM1, 1:500 (5114, Cell Signalling Technology, Danvers, Massachusetts, USA); LC3B, 1:500 (NB100-2220, Novus Biologicals, Centennial Colorado, USA); β-Actin, 1:5000 (A2228, Sigma-Aldrich, St. Louis, Missouri, USA); GAPDH, 1:5000 (MA5-15738, Invitrogen, Waltham, Massachusetts, USA). Secondary antibodies used were: Donkey anti-Rabbit IgG, 1:10,000 (NA943V, GE Healthcare, Chicago, Illinois, USA); Goat anti-Mouse IgG, 1:10,000 (A0168, Sigma-Aldrich, St. Louis, Missouri, USA).

## Results

### Generation of mice with inducible monoallelic deletion of *Becn1* in the intestinal epithelium

**Fig. 1:**
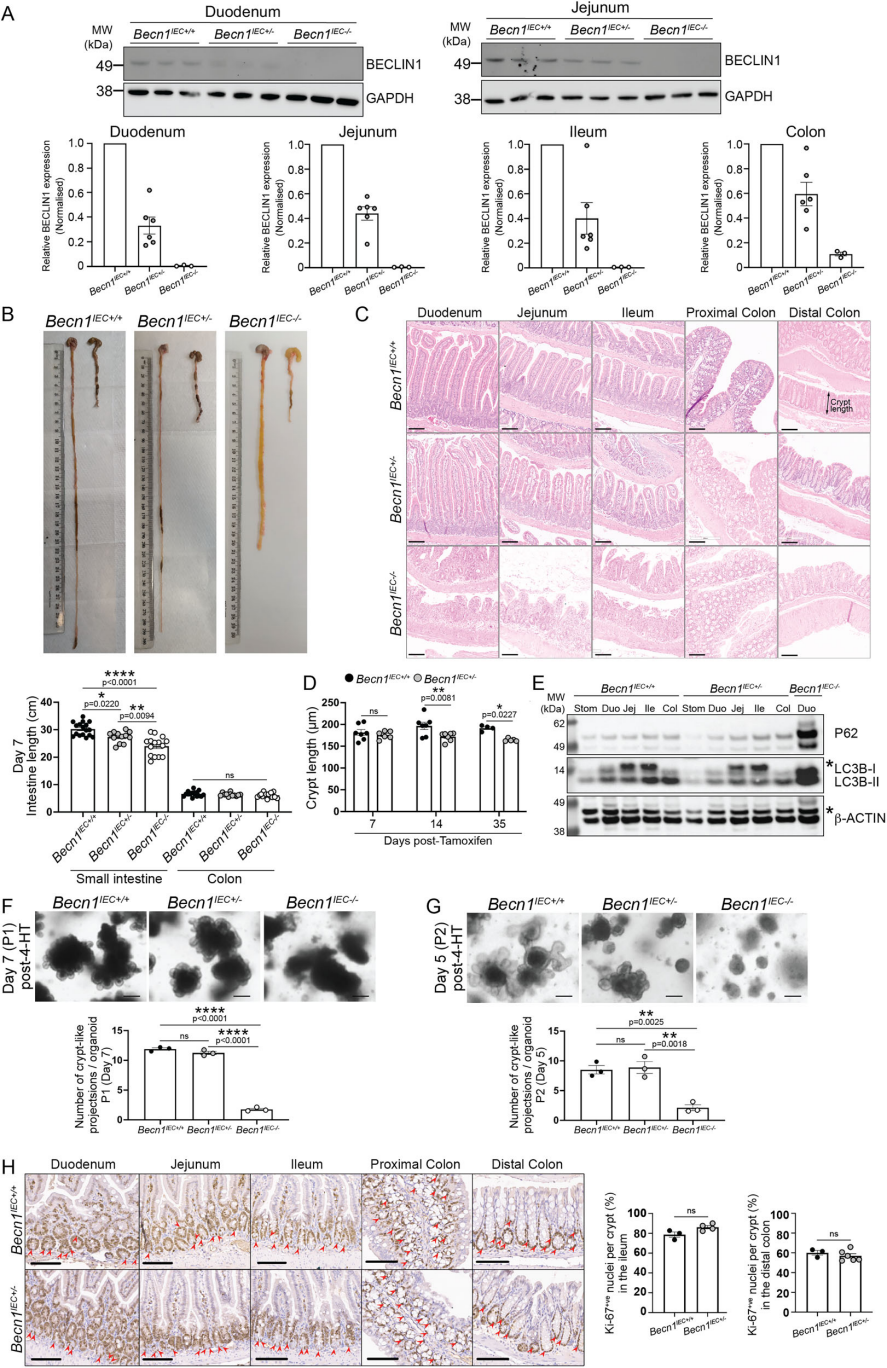
Monoallelic *Becn1* deletion induces mild intestinal epithelial alterations under basal conditions, distinct from the severe defects observed with homozygous *Becn1* deletion. **A** Representative Western blots (top) showing BECLIN1 levels in the indicated tissues, with densitometry quantification (bottom). To enable comparison across Western blots, BECLIN1 band volumes were quantified and expressed as BECLIN1:GAPDH ratios, then normalised to the mean BECLIN1:GAPDH ratio of the *Becn1*^*IEC+/+*^ group (set to 1.0). Data represent *n* = 6 for *Becn1*^*IEC+/+*^ and *Becn1*^*IEC+/-*^ mice and *n* = 3 for *Becn1*^*IEC-/-*^ mice. All blots showing all samples used in the quantification are in Supplementary Fig. 1B; GAPDH was used as a loading control. **B** Representative images of intestinal tracts of *Becn1*^*IEC+/+*^, *Becn*^*IEC+/-*^ and *Becn1*^*IEC+/+*^ mice (top) with the measurements of intestinal length at Day 7 post Tamoxifen administration (bottom). Data represent *n* = 12 to 17 animals per genotype from *n* > 3 independent experiments. **C** H&E-stained FFPE sections of the intestinal tracts from *Becn1*^*IEC+/+*^*, Becn1*^*IEC+/-*^ and *Becn1*^*IEC-/-*^ mice at Day 7 post Tamoxifen administration. Scale bars = 100 μm. Data represent *n* = 9 biologically independent mice of each genotype from *n* = 3 independent experiments. **D** Quantification of distal colon crypt length in *Becn1*^*IEC+/+*^ and *Becn1*^*IEC+/-*^ mice at days 7, 14 and 35 post-tamoxifen administration. Only visible full-length crypts were included in the analysis, with *n* > 5 crypts measured per mouse and averaged. Data represent *n* = 4 to 7 animals per genotype from *n* = 3 independent experiments. **E** Representative Western blot (single biological sample per genotype) showing markers of basal autophagy across different sections of the gastrointestinal tract in *Becn1*^*IEC+/+*^ and *Becn1*^*IEC+/-*^ mice at Day 7 post 4-HT treatment. The duodenum of *Becn1*^*IEC-/-*^ mice was included as a positive control for defective autophagy. β-ACTIN was used as a loading control. Asterisks denote non-specific bands arising from carryover on the previous membrane or from antibody cross-reactivity. **F** Representative phase contrast microscopy images of *Becn1*^*IEC+/+*^, *Becn1*^*IEC+/-*^ and *Becn1*^*IEC-/-*^ organoids at Day 7 post-4-HT treatment and **G** re-passaged surviving *Becn1*^*IEC+/+*^, *Becn1*^*IEC+/-*^ and *Becn1*^*IEC-/-*^ organoids at Day 5, along with respective quantification of the number of budding crypt-like projections per organoid. Scale bar = 100 µm. **H** Representative Ki-67 immunostaining of FFPE sections from *Becn1*^*IEC+/+*^ and *Becn1*^*IEC+/-*^ mice at Day 7 post-tamoxifen administration across different segments of the small and large intestine. Red arrowheads indicate intact proliferative activity within the intestinal stem cell compartments. Data represent *n* = 6 mice from *n* = 3 independent experiments. Scale bar = 100 μm. Accompanying graphs show quantification of Ki-67 staining expressed as the percentage of Ki-67^+ve^ nuclei per crypt across representative regions of the small (ileum) and large (distal colon) intestine. For each mouse, 3–5 crypts were analysed and averaged, with 3 to 6 mice per genotype included. Data in (**F**, **G**) were obtained from *n* = 20 organoids per genotype across *n* = 3 independent experiments. Graphs show the mean ± S.E.M. Statistical significance was determined by unpaired (Student’s) *t* test in (**A**, **D**, **H**), and ordinary one-way ANOVA in (**B**, **F**, **G**). SI small intestine, Stom stomach, Duo duodenum, Jej jejunum, Ile ileum, Col Colon, MW molecular weight, 4-HT 4-hydroxytamoxifen, ns not significant (*p* > 0.05).

We previously reported that homozygous *Becn1* deletion in the intestinal epithelium results in rapid, fatal enterocolitis, with severe epithelial disruption [23]. To model the haploinsufficiency seen in human disease where BECLIN1 is reduced, we generated mice in which monoallelic *Becn1* deletion could be induced in the intestinal epithelium using mice heterozygous for the *Becn1* floxed allele (*Becn1*^*fl/+*^*)* and carrying the *Vil1-Cre*^*ERT2*^ transgene (*Becn1*^*fl/+*^;*Vil1-CreERT2*^*Cre/+*^). Littermates lacking (*Becn1*^*+/+*^) or homozygous (*Becn1*^*fl/fl*^) for the floxed allele but positive for *Vil1-CreERT2*^*Cre/+*^ (*Becn1*^*+/+*^;*Vil1-CreERT2*^*Cre/+*^ or *Becn1*^*fl/fl*^;*Vil1-CreERT2*^*Cre/+*^ respectively) served as controls. Intraperitoneal Tamoxifen injections induced deletion in the small intestine (duodenum, jejunum, ileum) and colon of *Becn1*^*fl/+*^*;Vil1-CreERT2*^*Cre/+*^ mice, confirmed genomically and by protein analysis showing approximately 50% reduced BECLIN1 levels (Fig. 1A, Supplementary Fig. 1A, B). Tamoxifen-treated *Becn1*^*+/+*^;*Vil1-CreERT2*^*Cre/+*^, *Becn1*^*fl/+*^;*Vil1-CreERT2*^*Cre/+*^, and *Becn1*^*fl/fl*^;*Vil1-CreERT2*^*Cre/+*^ mice are hereafter respectively referred to as *Becn1*^*IEC+/+*^, *Becn1*^*IEC+/-*^ and *Becn1*^*IEC-/-*^ mice.

### Mice with reduced BECLIN1 have shortened small intestines and reduced colonic crypt lengths without other overt phenotypes

At 7 days post-BECLIN1 deletion, there were no significant body weight differences between *Becn1*^*IEC+/+*^ and *Becn1*^*IEC+/-*^ mice (Supplementary Fig. 2A), unlike the marked weight loss previously seen with homozygous *Becn1* deletion in the intestinal epithelium [23]. Despite appearing grossly normal, *Becn1*^*IEC+/-*^ mice displayed significantly shorter small intestines than *Becn1*^*IEC+/+*^ controls, while colon lengths were unchanged (Fig. 1B). Histological examination by H&E staining revealed preserved epithelial architecture in the small and large intestine of *Becn1*^*IEC+/-*^ mice, with intact villi, organised crypt structure, and no evidence of epithelial damage or inflammation at 7 days post deletion (Fig. 1C). At this time point, distal colonic crypt length (Fig. 1D) and width (Supplementary Fig. 2B) did not differ between genotypes. However, by 14- and 35-days post deletion, *Becn1*^*IEC+/-*^ mice exhibited significantly shorter colonic crypts and reduced crypt volume fraction [37] compared to controls (Fig. 1D). Observation over one month post deletion also revealed a persistently shorter small intestine (Supplementary Fig. 2C) without weight loss (Supplementary Fig. 2A), and otherwise normal intestinal morphology (Supplementary Fig. 2D). These phenotypes contrasted sharply with *Becn1*^*IEC-/-*^ mice, where small intestines were shortened, swollen, and lytic, with villus stunting along the entire length within 7 days of gene deletion (Fig. 1B, C) [23]. In summary, apart from reduced small intestinal and colonic crypt lengths, heterozygous *Becn1* deletion does not cause overt abnormalities under basal conditions up to one month post deletion, in contrast to its homozygous loss [23].

### Intestinal epithelial cells with reduced BECLIN1 levels display no disruption to basal autophagy or proliferation

Monoallelic *Becn1* deletion caused no dramatic changes in basal autophagy in a representative mouse where BECLIN1 protein levels were reduced (Fig. 1A), with P62 levels and LC3B (total and LC3B-I:LC3BII) levels appearing comparable to wild-type IECs at 7 days post deletion (Fig. 1E, Supplementary Fig. 1C). In contrast, *Becn1*^*IEC-/-*^ IECs showed pronounced increases in P62, total LC3B and LC3B-I:LC3BII (Fig. 1E, Supplementary Fig. 1C) [23]. We previously identified an essential role for BECLIN1 in endocytic trafficking in small intestinal-derived organoids [22, 23]. To test whether monoallelic *Becn1* loss induced similar defects, we generated small intestinal organoids from *Becn1*^*+/+*^; *Becn1*^*fl/+*^; and *Becn1*^*fl/fl*^;*Vil1-CreERT2*^*Cre/+*^ mice. Treatment with 4-hydroxytamoxifen (4-HT) led to reduced BECLIN1 expression in *Becn1*^*IEC+/-*^ organoids (Supplementary Fig. 3A) which did not impact basal autophagy (as with intestinal epithelial cells from mice, Supplementary Fig. 3B), unlike homozygous *Becn1* deletion.

At 7 days post 4-HT treatment, *Becn1*^*IEC-/-*^ organoids showed significantly reduced budding crypt formation (Fig. 1F) [23], whereas *Becn1*^*IEC+/-*^ organoids formed crypt-like projections comparable to *Becn1*^*IEC+/+*^ organoids (Fig. 1F). While budding morphology alone cannot establish proliferation or differentiation status, this suggests that gross organoid growth capacity is preserved in the heterozygous setting. Re-passaging of *Becn1*^*IEC+/+*^ and *Becn1*^*IEC+/-*^ cultures yielded mature organoids by Day 5, with similar numbers of budding crypts (Fig. 1G). Importantly, this is consistent with Ki-67 staining of intestinal tissues from *Becn1*^*IEC+/-*^ mice, which showed no significant change in the percentage of Ki-67^+ve^ nuclei per crypt compared to controls (Fig. 1H). In contrast, surviving *Becn1*^*IEC-/-*^ organoids failed to generate viable organoid structures upon re-passaging, producing significantly fewer crypt-like projections by Day 5 (Fig. 1G). Together, these findings indicate that reduced BECLIN1 expression does not impair basal autophagy or epithelial cell survival, and proliferation remains largely intact in the heterozygous state.

### Intestinal epithelial cells with reduced BECLIN1 levels alters endocytic trafficking, E-CADHERIN distribution, and actin-dependent epithelial architecture

**Fig. 2:**
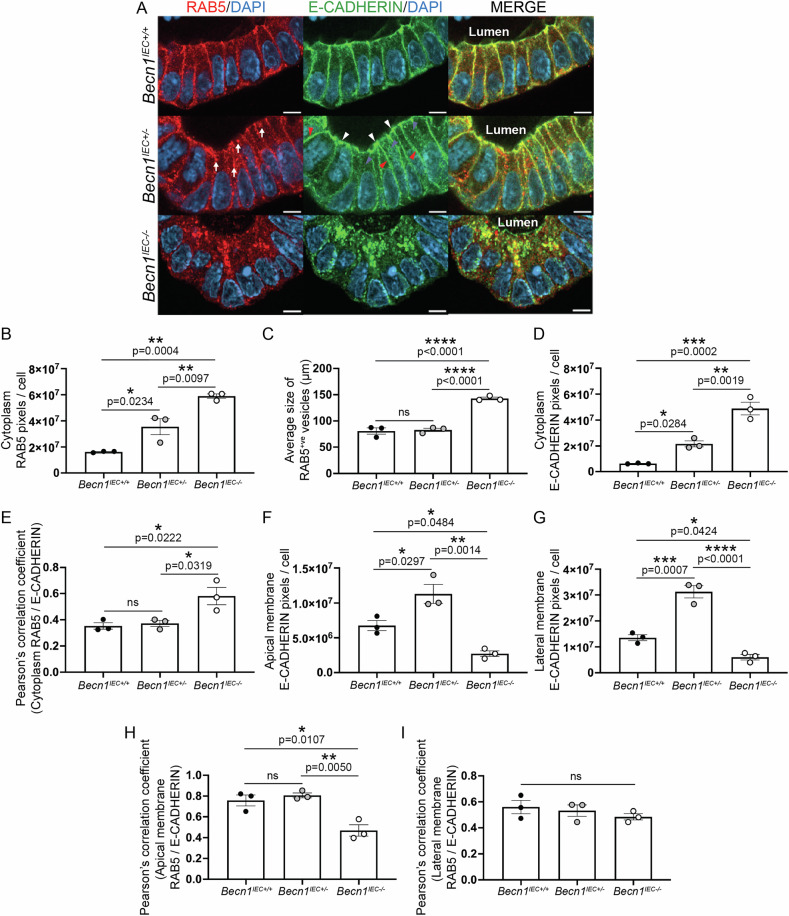
BECLIN1 reduction alters E-CADHERIN trafficking in a RAB5-independent manner. **A** Representative whole-mount immunofluorescence staining for RAB5 (red), E-CADHERIN (green) and DAPI (blue) in *Becn1*^*IEC+/+*^*, Becn1*^*IEC+/−*^*and Becn1*^*IEC−/−*^ organoids. Increased cytoplasmic RAB5 is indicated by white arrows. Aberrant E-CADHERIN localisation is denoted by white (apical), red (lateral), and purple (cytoplasmic) arrowheads. Scale bar = 5 μm. **B** Quantification of cytoplasmic RAB5 pixels per cell. **C** Measurement of the average size of RAB5^+ve^ vesicles. **D** Quantification of cytoplasmic E-CADHERIN pixels per cell, along with its (**E**) co-localisation with cytoplasmic RAB5, measured using Pearson’s correlation coefficient. Quantification of (**F**) apical and (**G**) lateral E-CADHERIN pixels per intestinal epithelial cell. Measurement of the degree of colocalisation between RAB5 and E-CADHERIN on the (**H**) apical and (**I**) lateral membrane of intestinal epithelial cells. Data are representative of at least *n* = 3 different slices per organoid and of at least *n* = 3 biologically independent organoids. For each z-section, at least *n* = 3 individual IECs with clear apical to basal delineation were used. Graphs indicate the mean ± S.E.M. Statistical significance was determined using ordinary one-way ANOVA. ns not significant (*p* > 0.05).

We previously reported that homozygous BECLIN1 deletion in small intestinal epithelial cells (IECs) caused pronounced trafficking defects that compromise barrier integrity *via* mislocalisation of junctional proteins [22, 23, 38–42]. To determine whether this also occurs under heterozygous conditions, we examined *Becn1*^*IEC+/−*^ small intestinal-derived organoids. Compared with *Becn1*^*IEC+/+*^, *Becn1*^*IEC+/−*^ organoids displayed a modest but significant increase in cytoplasmic RAB5^+ve^ early endosomes (Fig. 2A, B, Supplementary Fig. 4A) though without the aberrant enlargement seen in *Becn1*^*IEC-/-*^ organoids (Fig. 2A, C). In the homozygous setting, the junctional protein E-CADHERIN is sequestered within the enlarged cytoplasmic RAB5^+ve^ early endosomes (Fig. 2A, D, E) [23]. However, in *Becn1*^*IEC+/−*^ IECs, E-CADHERIN levels were instead increased at apical and lateral membranes (Fig. 2F, G) and in the cytoplasm (Fig. 2D), but notably, with no change in RAB5 co-localisation (Fig. 2E, H, I). Thus, reduced BECLIN1 alters early endosomal dynamics, but E-CADHERIN localisation changes appear independent of RAB5-mediated trafficking.

**Fig. 3:**
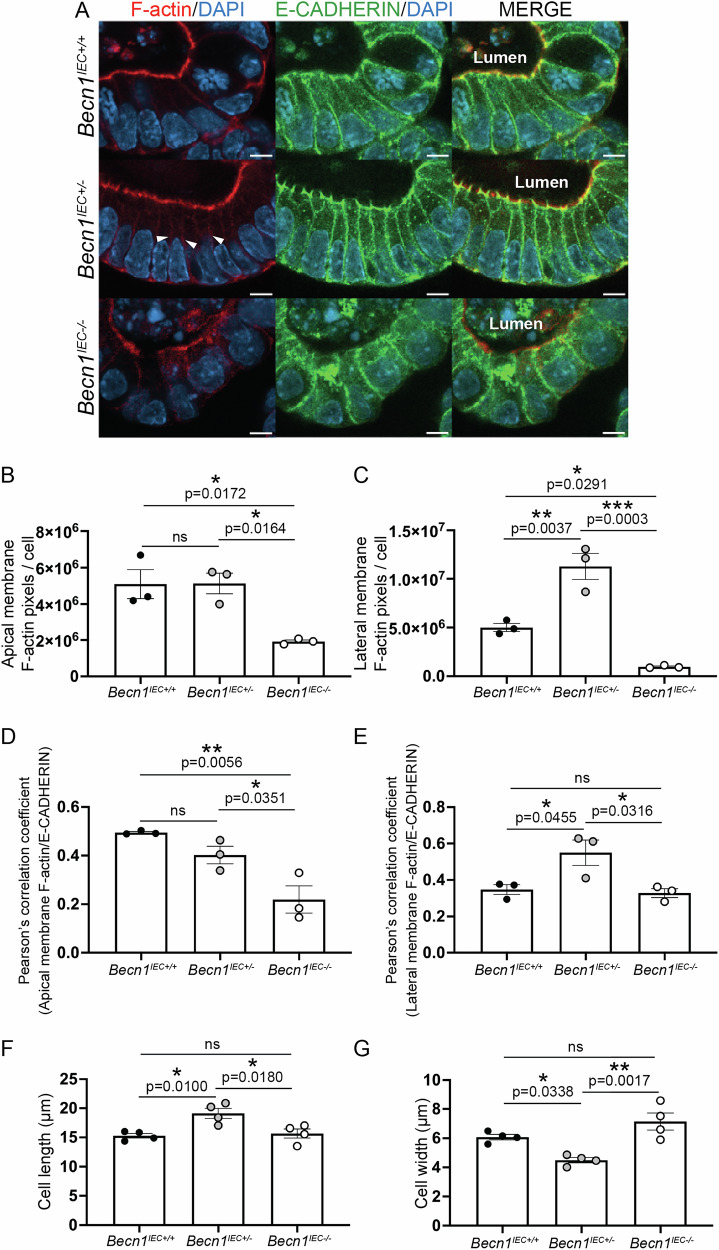
Monoallelic *Becn1* deletion alters F-actin organisation in intestinal epithelial cells. **A** Representative whole-mount immunofluorescence staining for F-actin (red), E-CADHERIN (green) and DAPI (blue) in *Becn1*^*IEC+/+*^*, Becn1*^*IEC+/−*^*and Becn1*^*IEC−/−*^ organoids. Increased lateral F-actin in *Becn1*^*IEC+/−*^ organoids is indicated by white arrowheads. Scale bar = 5 μm. Quantification of (**B**) apical and (**C**) lateral F-actin signals per cell. Colocalisation between F-actin and E-CADHERIN on the (**D**) apical and (**E**) lateral membranes of intestinal epithelial cells, assessed using Pearson’s correlation coefficient. **F** Measurement of cell length (apical to basal membrane) and (**G**) cell width (lateral to lateral membrane). Data are representative of at least *n* = 3 different z-slices per organoid and of at least *n* = 3 biologically independent organoids. For each z-slice, at least *n* = 3 individual cells with clear apical to basal delineation were used for quantification. Graphs indicate the mean ± S.E.M. Statistical significance was determined using ordinary one-way ANOVA. ns not significant (*p* > 0.05).

Cytoskeletal remodelling can influence cadherin localisation [43]. Consistent with this, we previously showed that homozygous BECLIN1 loss disrupts the cytoskeleton and compromises the intestinal epithelial barrier [22]. Therefore, we next examined F-actin organisation, which is critical for adhesion and endocytosis [44, 45]. *Becn1*^*IEC+/−*^ organoids exhibited increased F-actin, and co-localised E-CADHERIN, along the lateral membrane but not apical membrane (Fig. 3A–E), consistent with an F-actin-driven “cadherin flow” mechanism, whereby cadherins are transported along remodelling actin networks [46]. Notably, E-CADHERIN localisation was redistributed within the lateral membrane toward apicolateral junctions (Fig. 3E), potentially reinforcing cell-cell adhesion and the preservation of epithelial integrity. Given this increased lateral membrane localisation of both E-CADHERIN and F-actin in *Becn1*^*IEC+/-*^ IECs (Figs. 2G, 3C, E), *Becn1*^*IEC+/−*^ organoids had a longer apico-basal axis and shorter IEC width (Fig. 3A, F, G, Supplementary Fig. 4B), likely contributing to the shorter small intestinal length (Fig. 1B). Reduced BECLIN1 therefore alters early endosomal dynamics and F-actin-guided adhesion remodelling, leading to compensatory redistribution of junctional proteins and changes in epithelial architecture.

### Monoallelic *Becn1* deletion in the intestinal epithelium causes goblet cell defects

**Fig. 4:**
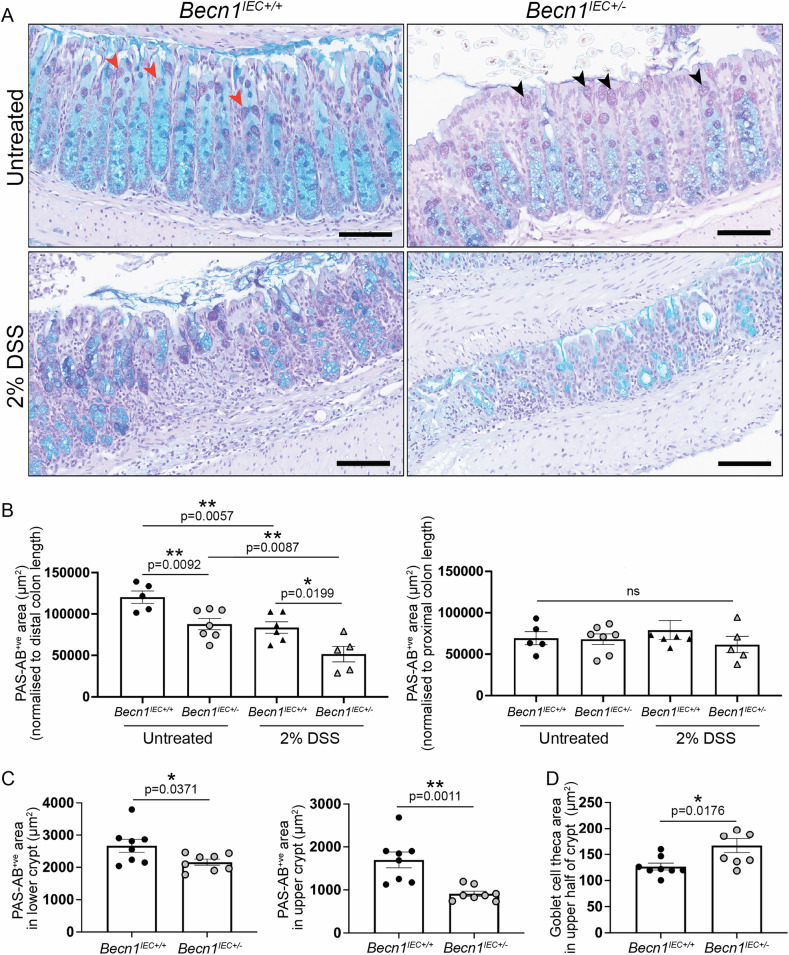
Monoallelic *Becn1* deletion leads to goblet cell defects. **A** Representative PAS-AB-stained distal colon sections from untreated and DSS-treated *Becn1*^*IEC+/+*^ and *Becn1*^*IEC+/−*^ mice. Scale bar = 100 μm. Black arrowheads depict enlarged goblet cells in *Becn1*^*IEC+/−*^ mice compared with *Becn1*^*IEC+/+*^ goblet cells indicated with orange arrowheads. Data represent *n* = 9 biologically independent mice of each genotype from *n* = 3 independent experiments. **B** Quantification of PAS-AB^+ve^ area (measured per µm of muscularis mucosae) normalised to gross colon length in the distal and proximal colon of untreated and DSS-treated *Becn1*^*IEC+/+*^ and *Becn1*^*IEC+/−*^ mice. Data represent *n* = 5 to 7 biologically independent mice per genotype from *n* = 3 independent experiments. **C** Quantification of PAS-AB^+ve^ area in the lower and upper colonic crypt compartments, as delineated in Supplementary Fig. 5B, in *Becn1*^*IEC+/+*^ and *Becn1*^*IEC+/-*^ mice. Data represent *n* = 8 animals per genotype from *n* = 3 independent experiments. For each mouse, 3–6 crypts were analysed and averaged. **D** Quantification of goblet cell theca size in the upper colonic crypt compartments of *Becn1*^*IEC+/+*^ and *Becn1*^*IEC+/-*^ mice. Data represent *n* = 7 to 8 biologically independent mice per genotype from *n* = 2 independent experiments. Graphs show the ± S.E.M. Statistical significance was determined by ordinary one-way ANOVA in (**B)** and unpaired (Student’s) t-test in (**C**, **D**). For crypt-specific quantifications in (**C**), full-length intact crypts with at least three contiguous intact crypts were analysed, with *n* > 3 crypts analysed per animal. The average goblet cell theca area in (**D**) was obtained by measuring >100 goblet cells per animal. Representative images used for quantification shown in Fig. 4B–D are shown in Supplementary Fig. 5A. DSS dextran sulphate sodium, PAS periodic acid Schiff, AB alcian blue, ns not significant (*p* > 0.05).

Complete loss of BECLIN1 in the intestinal epithelium causes severe, spontaneous enteritis originating in the small intestine [23]. Accordingly, the fatal nature of this phenotype, and the need for early euthanasia limited assessment of whether the colon is similarly affected. In the heterozygous setting, however, we observed no spontaneous small intestinal inflammation. Instead, from 14 days post gene deletion onwards (Fig. 1D), *Becn1*^*IEC+/-*^ mice exhibited shortened colonic crypts, suggesting that the colon may represent a site of vulnerability to partial BECLIN1 loss over time. Guided by this observation, we next examined the colonic epithelium more closely. Periodic-Acid-Schiff (PAS) and Alcian Blue (AB) staining revealed a significant reduction in PAS-AB staining in the distal colon (Fig. 4A - top panels, B, Supplementary 5A), but not the proximal colon (Fig. 4B), of *Becn1*^*IEC+/-*^ mice compared to *Becn1*^*IEC+/+*^ mice (Fig. 4A, B, Supplementary Fig. 5A), suggesting reduced goblet cell-associated mucin content and/or goblet cell abundance [13]. As goblet cell maturation involves migration to the crypt surface, we also quantified PAS-AB^+ve^ mucin area in the lower (immature) *versus* upper (mature) colonic crypt compartments (Fig. 4C, Supplementary Fig. 5B) [13, 47]. PAS-AB^+ve^ mucin staining was reduced in both lower and upper crypt compartments, but more so in the latter (50% versus 15%) (Fig. 4C). This maturation defect was supported by the enlarged cytoplasmic mucin (theca) areas in the upper crypt compartments of remaining mature goblet cells of *Becn1*^*IEC+/-*^ mice compared with *Becn1*^*IEC+/+*^ mice (Fig. 4A, D), consistent with impaired mucin secretion. These results show that reduced BECLIN1 results in reduced goblet cell-associated mucin content and defective mucin production which can potentially compromise mucosal barrier function.

### Reduced BECLIN1 levels in intestinal epithelial cells increases susceptibility to DSS-induced colitis

**Fig. 5:**
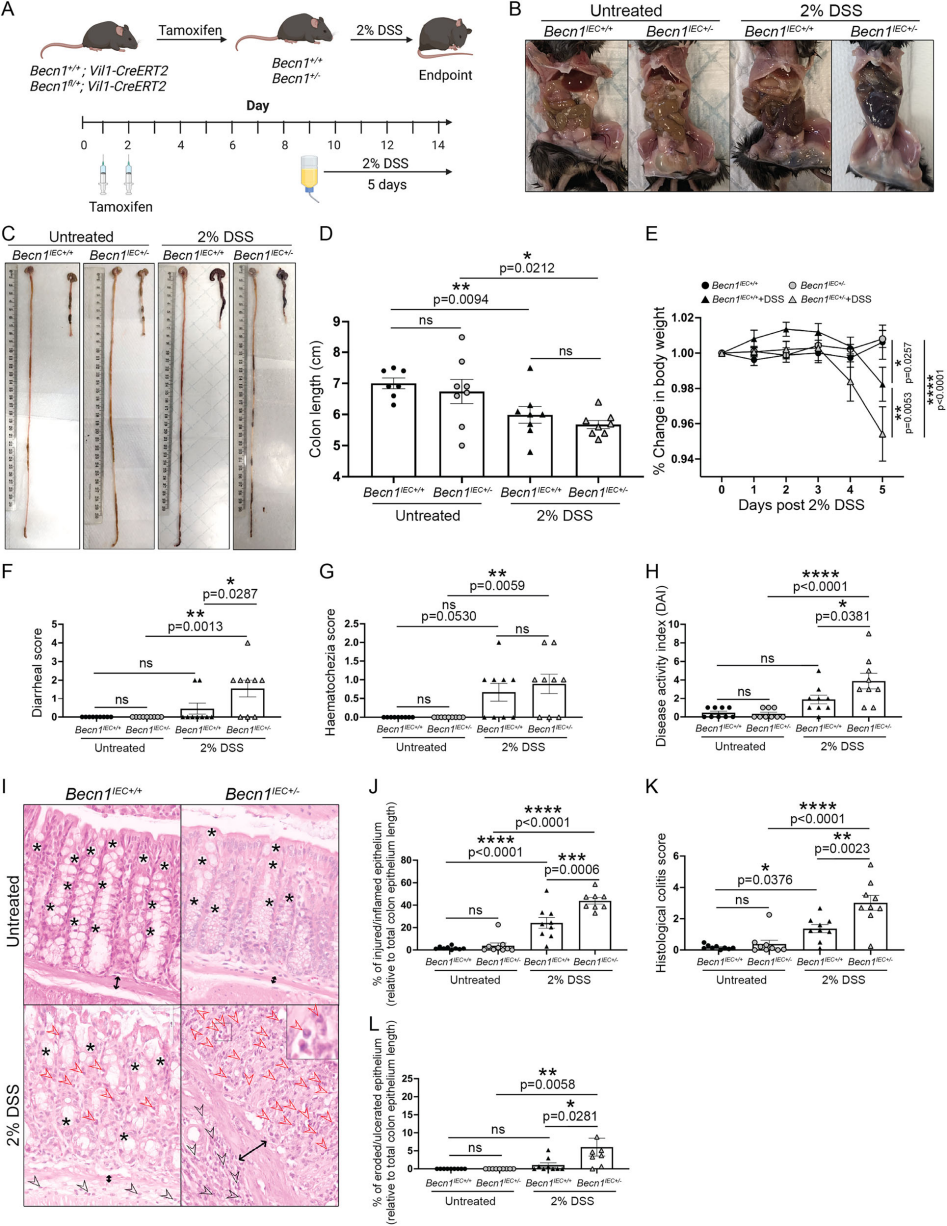
Reduced levels of BECLIN1 in the intestinal epithelium predisposes to Dextran Sulphate Sodium-induced colitis. **A** Schematic diagram depicting the timeline of Tamoxifen-induced *Becn1* gene deletion followed by 5 days of 2% Dextran Sulphate Sodium (2% DSS) treatment. Image created in BioRender.com. **B** Representative images of abdominal necropsy and **C** intestinal tracts of *Becn1*^*IEC+/+*^ and *Becn1*^*IEC+/−*^ mice, who received normal drinking water (untreated) or 2% DSS drinking water, at endpoint. **D** Colon lengths of mice from each treatment group at endpoint. **E** Changes in body weight of *Becn1*^*IEC+/+*^ and *Becn1*^*IEC+/−*^ mice during the 2% DSS treatment period, normalised to body weight at the start of DSS treatment (day 9). **F** Diarrhoeal and (**G**) haematochezia scores of *Becn1*^*IEC+/+*^ and *Becn1*^*IEC+/−*^ mice who received either normal drinking water or 2% DSS drinking water. Scores were determined based on parameters outlined in Table 1. **H** Disease Activity Index (DAI) score of *Becn1*^*IEC+/+*^ and *Becn1*^*IEC+/−*^ mice receiving either normal drinking water or 2% DSS drinking water, calculated by summing the scores of the parameters outlined in Table 1. **I** H&E-stained FFPE distal colon sections from *Becn1*^*IEC+/+*^ and *Becn1*^*IEC+/−*^ mice who received normal drinking water (untreated) or 2% DSS drinking water. The black double-headed arrow denotes the muscularis mucosae, and goblet cells are indicated by *. Immune cell infiltration in the submucosa and mucosa is marked by black and red open arrowheads, respectively. A magnified area (indicated by the box) provides a closer view of the mucosa, illustrating the increased immune infiltration in DSS-treated *Becn1*^*IEC+/−*^ mice. **J** Percentage of injured or inflamed epithelium, normalised to the length of the muscularis mucosae, in the colon of *Becn1*^*IEC+/+*^ and *Becn1*^*IEC+/−*^ mice receiving either normal drinking water or 2% DSS drinking water. **K** The histological colitis scores (HCS) of *Becn1*^*IEC+/+*^ and *Becn1*^*IEC+/−*^ mice with and without DSS treatment was calculated based on described methods [55]. **L** Percentage of eroded epithelium, normalised to the length of the muscularis mucosae, in the colon of *Becn1*^*IEC+/+*^ and *Becn1*^*IEC+/−*^ mice receiving either normal drinking water or 2% DSS drinking water. Data are representative of *n* = 7 to 9 biologically independent mice per genotype and treatment group, from *n* = 3 independent experiments in (**D**, **F**, **G**, **H**, **J**, **K**, **L**). Graphs indicate the ± S.E.M. Statistical significance was determined using ordinary one-way ANOVA except in (**E**) where changes in body weight were determined using two-way ANOVA with Tukey’s post-hoc test. Images in (**B**, **C**, **I**) are representative of at least *n* = 7 to 9 biologically independent mice from *n* = 3 independent experiments.

Goblet cells are secretory IECs specialised for mucus production, forming a critical barrier against pathogens and mechanical damage [48]. Disruption of this barrier is a hallmark of chronic inflammatory conditions, including IBD, often arising from goblet cell loss or defects in mucin formation, storage, or secretion [48–51]. To test whether the goblet cell defects seen in *Becn1*^*IEC+/−*^ mice increase susceptibility to inflammation, 7- to 14-week old mice were treated with dextran sulphate sodium (DSS) to induce colitis, a widely used model of IBD pathogenesis [52]. Following Tamoxifen-induced gene deletion, mice received either 2% DSS in drinking water for 5 days or normal water (Fig. 5A). There were no genotype-dependent differences in starting body weight or DSS-water consumption (Supplementary Fig. 6A, B).

At endpoint, both DSS-treated genotypes displayed expected colitic changes, including darker, reddish areas in the colon consistent with hyperaemia/inflammation (Fig. 5B, C) and colon shortening with swelling and constriction (Fig. 5C, D), but gross colonic changes were similar (Fig. 5B–D). The small intestines of *Becn1*^*IEC+/−*^ mice were significantly shorter than *Becn1*^*IEC+/+*^ controls regardless of DSS treatment (Supplementary Fig. 6C). Both genotypes lost weight after DSS exposure, but this was greater in *Becn1*^*IEC+/−*^ mice (Fig. 5E). They also developed more severe diarrhoea, with a higher proportion producing pasty to liquid stools (Fig. 5F), though hematochezia did not differ (Fig. 5G). Importantly, the composite disease activity index (DAI), incorporating weight loss, stool consistency, and rectal bleeding (Table 1) [53, 54], was significantly higher in *Becn1*^*IEC+/−*^ mice, indicating greater colitis severity (Fig. 5H).

Histological assessment of DSS-treated colons was performed using a blinded scoring system evaluating crypt architecture, inflammatory cell infiltration, and epithelial integrity, with the composite score reported as the histological colitis score (HCS) [55]. Untreated mice of both genotypes had minimal mucosal damage and low histological colitis scores (HCS) (Fig. 5I, J, K). DSS treatment increased the HCS scores in both genotypes, but *Becn1*^*IEC+/−*^ mice scored approximately 2.2-fold higher (Fig. 5K), with more severe epithelial injury and crypt attenuation, epithelial loss, and immune infiltration (Fig. 5I, J). These mice also had more regions of epithelial erosion (Fig. 5L), characterised by complete crypt loss and dense localised immune cell infiltration. Lymphoid follicle counts and sizes did not differ between groups (Supplementary Fig. 6D, E), indicating that inflammation was largely confined to the epithelial layer. Hence, monoallelic *Becn1* loss worsens DSS-induced colitis, indicating increased sensitivity to intestinal inflammation.

### Reduced BECLIN1 levels disrupt neutral mucins at baseline and exacerbates mucin loss following DSS treatment

**Fig. 6:**
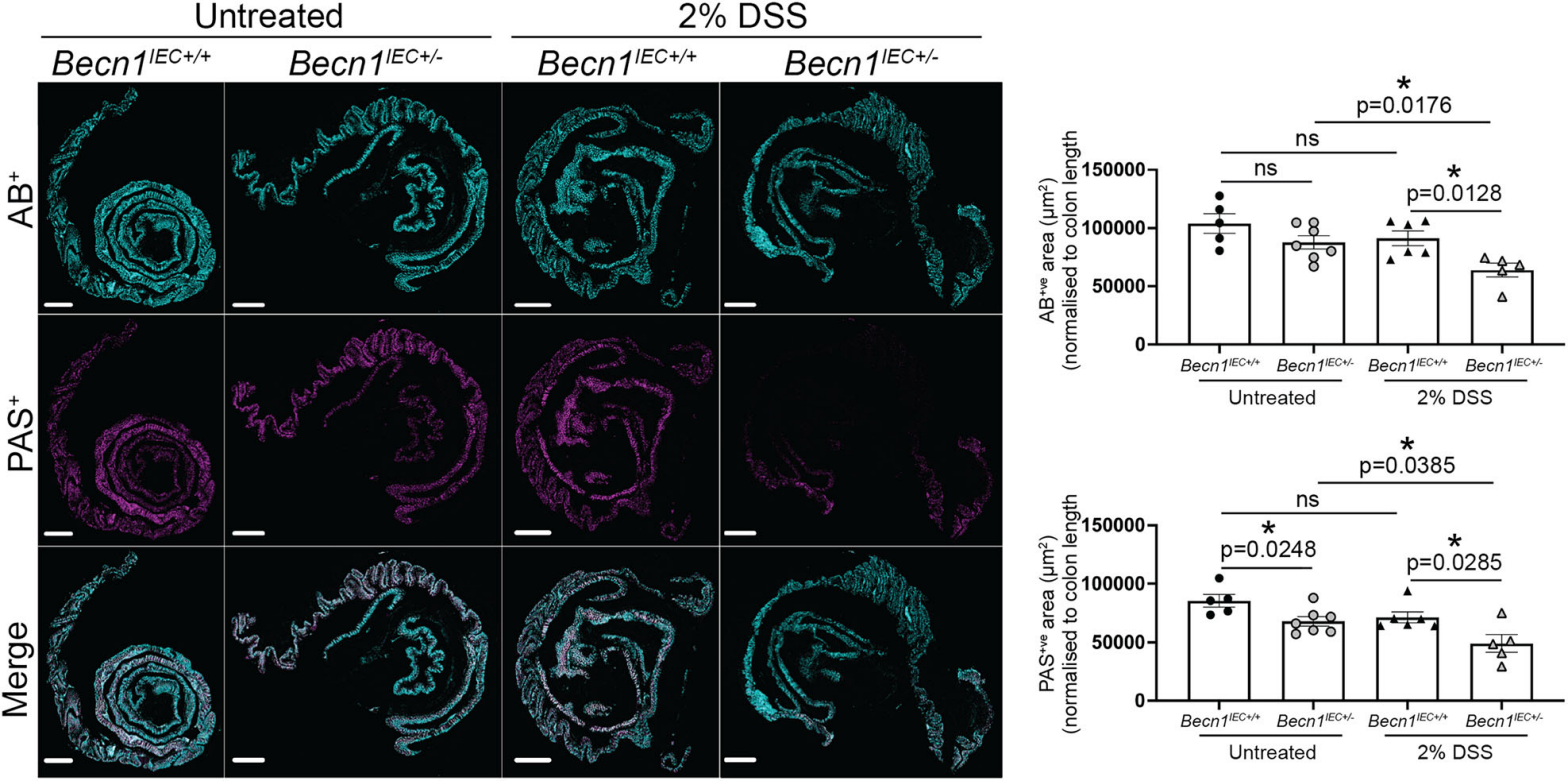
Reduced BECLIN1 levels disrupt neutral mucins at baseline and exacerbates mucin loss following DSS treatment. Representation of acidic (AB^+^), neutral (PAS^+^), and total (Merge) mucins in untreated and DSS-treated *Becn1*^*IEC+/+*^ and *Becn1*^*IEC+/−*^ mice. Scale bars = 1 mm. The areas of acidic (AB^+ve^) and neutral (PAS^+ve^) mucus were quantified per µm of muscularis mucosae and normalised to gross total colon length. Data are representative of *n* = 5 to 7 biologically independent mice per genotype and treatment group, from *n* = 2 independent experiments. Graphs show the ±S.E.M. Statistical significance was determined by ordinary one-way ANOVA. Images are representative of at least *n* = 5 to 7 biologically independent mice from *n* = 2 independent experiments. Only animals that received DSS treatment from the same lot number were included for analysis. DSS dextran sulphate sodium, PAS periodic acid Schiff, AB alcian blue, ns not significant (*p* > 0.05).

We next investigated goblet cell responses in DSS-treated *Becn1*^*IEC+/+*^ and *Becn1*^*IEC+/−*^ mice. The epithelial damage in both DSS-treated *Becn1*^*IEC+/+*^ and *Becn1*^*IEC+/−*^ mice (Fig. 5I–L) was accompanied by a significant reduction in PAS-AB^+ve^ mucin area in the distal colon compared with their respective untreated controls (Fig. 4A, B), consistent with the distal colon being the primary site of DSS-induced injury [56]. Notably, the epithelial damage in the distal colon was exacerbated in DSS-treated *Becn1*^*IEC+/−*^ mice, where the PAS-AB^+ve^ mucin area was significantly reduced when compared with DSS-treated *Becn1*^*IEC+/+*^ mice (Fig. 4A, B, Supplementary Fig. 5A). We also assessed PAS-AB staining across the entire colon, which revealed a loss of PAS^+ve^ neutral mucins in *Becn1*^*IEC+/−*^ mice compared to *Becn1*^*IEC+/+*^ in the untreated groups, with no significant change in AB^+ve^ acidic mucins (Fig. 6). Notably, following DSS treatment, unlike *Becn1*^*IEC+/+*^ mice, *Becn1*^*IEC+/−*^ mice exhibited a loss of both neutral and acidic mucins, consistent with the exacerbated epithelial damage observed (Figs. 5I–L, 6). This reveals a vulnerability of the mucosal barrier when BECLIN1 is reduced, reinforcing its role in epithelial homoeostasis. The selective baseline loss of neutral mucins, and subsequent loss of both mucin types after DSS treatment, highlights the critical importance of neutral mucins in protecting against DSS-induced colitis.

## Discussion

We previously identified an essential role for BECLIN1 in maintaining intestinal homoeostasis and barrier integrity mediated through its autophagy and endocytic trafficking functions [22, 23]. Given that homozygous *Becn1* deletion causes fatal small intestinal enteritis in adult mice [23], we investigated whether partial reduction, mimicking clinically relevant haploinsufficiency, also impairs intestinal function. Accordingly, this study is specifically focused on the consequences of haploinsufficient BECLIN1 expression, as the rapid and fatal small intestinal pathology associated with homozygous deletion precludes detailed analysis of colonic phenotypes in that setting [23].

Unlike homozygous BECLIN1 deletion, monoallelic loss did not trigger overt spontaneous disease. However, *Becn1*^*IEC+/−*^ mice did display subclinical abnormalities, including shortened small intestines and alterations in epithelial cell morphology and organisation. Organoid studies demonstrated modest increases in cytoplasmic RAB5^+ve^ early endosomes but no enlargement, indicating that trafficking disruptions in the heterozygous state fall below the threshold seen in homozygous deletion [22, 23]. Instead changes in E-CADHERIN and F-actin distribution suggested cytoskeletal remodelling and altered cell shape, likely representing adaptive mechanisms to preserve junctional integrity, rather than explicit barrier failure. These data reinforce the idea that the small intestine is the primary site of overt pathology in homozygous deletion, whereas heterozygous loss is tolerated in this compartment under baseline conditions.

By contrast, the novel finding of this study is the identification of colonic goblet cell defects in *Becn1*^*IEC+/−*^ mice, a phenotype that was not apparent in the homozygous setting due to the rapid onset of fatal small intestinal disease precluding assessment of colonic changes [23]. Goblet cells were disproportionately affected by partial BECLIN1 loss, with reduced numbers, altered maturation (larger thecae), and diminished mucin staining, especially in the upper crypt. This aligns with the known role of autophagy in promoting goblet cell function via ER stress alleviation and mucin handling [11]. Given the central role of goblet cells and mucins in maintaining the mucus barrier, this baseline defect highlights the colon as a site of vulnerability to partial BECLIN1 loss. Following DSS challenge, *Becn1*^*IEC+/−*^ mice failed to mount the same level of compensatory mucin response as wild-type controls, showing depletion of both neutral and acidic mucins. The baseline vulnerability due to selective mucin loss, compounded by the broad depletion after DSS treatment, points to a barrier-specific weakness rather than uncontrolled inflammation, consistent with goblet cell dysfunction as a driver. These findings underscore the importance of neutral mucins as a critical buffer against colitis, in line with prior findings from MUC2-deficient mice [51] and mucin abnormalities in IBD patients [57–62]. They also extend current knowledge by positioning BECLIN1 insufficiency as a mechanism that selectively undermines goblet cell function and mucosal defence.

Although IBD GWAS have not identified BECLIN1 single nucleotide polymorphisms (SNPs), emerging evidence implicates both IEC-intrinsic and -extrinsic mechanisms that lower BECLIN1 levels, thereby exacerbating colitis. For instance, cyclic GMP-AMP synthase (cGAS) promotes autophagy in IECs through interaction with BECLIN1, and its loss worsens colitis [63]. Similarly, the m^6^A nuclear reader, YTHDC1, stabilises *Becn1* mRNA and is downregulated in DSS colitis and in IBD patient microbiota [35, 64]. Moreover, while no IBD-associated SNPs have been identified within *Becn1* itself, variants in upstream regulators such as STAT3, a transcriptional regulator of *Becn1*, have been implicated in IBD pathogenesis [65–67]. Caspase-mediated cleavage of BECLIN1 during apoptosis may also contribute to reduced protein levels in inflamed tissue, amplifying epithelial injury [68, 69]. Looking forward, resources such as atlases of IBD patient samples [70, 71] provide opportunities to examine BECLIN1 expression at endoscopically defined disease margins. Expanding clinical analyses will be critical to determine whether there are changes in BECLIN1 levels across broader clinical cohorts and to assess its potential as a biomarker of IBD pathogenesis and severity.

In summary, we show that while heterozygous BECLIN1 loss is tolerated in the small intestine under baseline conditions, it creates a colonic vulnerability characterised by goblet cell dysfunction and mucin depletion, sensitising the mucosa to DSS-induced colitis. These findings establish BECLIN1 as a critical epithelial defence regulator, with implications for stratifying patients by barrier vulnerability and guiding precision therapeutic interventions in IBD.

## Supplementary information

Supplemental Information

Original Data

## Data Availability

Correspondence and requests for materials should be addressed to Erinna F. Lee or Walter D. Fairlie.
