# Supplementary material for: Impact of BECLIN1 haploinsufficiency on goblet cell function and susceptibility to colitis
Source: Cell Death Dis. 2026 Jun 17;17(1):637. doi: 10.1038/s41419-026-08984-8 (PMC13365605; doi:10.1038/s41419-026-08984-8)
Supplement: Supplementary file 1 — Supplemental Information [file 41419_2026_8984_MOESM1_ESM.pdf]

SUPPLEMENTARY INFORMATION

MATERIALS AND METHODS

PCR genotyping

For colony maintenance and gene deletion assessments, DNA was extracted from ear clips or isolated IECs (see IEC isolation method). Samples were incubated overnight at 56°C in DirectPCR (Tail) Lysis Reagent (102-T, Viagen Biotech Inc., Los Angeles, California, USA) supplemented with 0.2% (v/v) Proteinase K (1.07393, Sigma-Aldrich, St. Louis, Missouri, USA), followed by heat inactivation at 85 °C for 1 hour. PCR amplification was performed using GoTaq PCR Master Mix (M7122, Promega, Madison, Wisconsin, USA) according to the manufacturer's protocol. Primers for *Becn1* reactions were as follows: "Beclin floxed forward" - 5'CTG ATC CTG CAG CTT GCA GAT TAG C3', "Beclin floxed reverse" - 5'CAC CAC TGC CTG GCT AAA CAA GAG C3', "Beclin KO reverse"- 5'CTA TAG AAG AAA GGA CTG TTG TGA AC3'. Primers for *Vil1-CreERT2* reactions are as follows: F: 5'CAA GCC TGG CTC GAC GGC C3', R: 5'CGC GAA CAT CTT CAG GTT CT3'. All primers were purchased from Integrated DNA Technologies as single stranded sequences purified by standard desalting and were resuspended at 10 OD/ml in nuclease-free H2O as working stock solutions. Reactions were cycled as follow: *Becn1* (Step 1: 94° C for 4 minutes, Step 2: 94° C for 30 seconds, Step 3: 55° C for 30 seconds, Step 4: 72° C for 1 minute, Step 5: Repeat steps 2-4 for an additional 29 times, Step 6: 72° C for 5 minutes, Step 7: Hold at 4° C) and *Vil1-CreERT2* (Step 1: 95° C for 5 minutes, Step 2: 94° C for 45 seconds, Step 3: 62° C for 25 seconds, Step 4: 72° C for 1.15 minute, Step 5: Repeat steps 2-4 for an additional 34 times, Step 6: 72° C for 5 minutes, Step 7: Hold at 4° C). Reaction products were electrophoresed on 1.5% (w/v) agarose gels containing SYBR™ Safe DNA

Gel Stain (S33102, Invitrogen™, Waltham, Massachusetts, USA) diluted at 20 000x in TAE buffer (40mM Tris Base, 20mM glacial acetic acid, 1mM EDTA). In all gels, 1 Kb Plus DNA Ladder (10787018, Invitrogen™, Waltham, Massachusetts, USA) was used as a marker for DNA migration according to size. DNA was visualised *via* UV excitation using the ChemiDoc™ Imaging System (Bio-Rad Laboratories, Hercules, California, USA) and images processed with Image Lab (Bio-Rad Laboratories, Hercules, California, USA).

Quantitative image analysis

Crypt length was quantified only in well-oriented, intact crypts in tissue sections with clearly defined luminal surfaces and crypt bases. Measurements were performed using Fiji/ImageJ by drawing a straight line along the longitudinal axis of the crypt from the luminal surface to the deepest point of the crypt base. Crypt width was measured by drawing a line perpendicular to the crypt length axis at the mid-crypt region, spanning from one lateral edge of the crypt to the opposite edge. All measurements were recorded in micrometers (μm), and values from multiple crypts per mouse were averaged for statistical significance.

Ki-67 immunohistochemistry was performed on formalin-fixed, paraffin-embedded intestinal sections using standard protocols. The ileum and distal colon were analysed as representative regions of the small and large intestine, respectively. Quantification was carried out on well-oriented intact crypts with clearly defined crypt bases and luminal surfaces. For each crypt, a region of interest encompassing the full crypt epithelium was defined, and the total number of nuclei and Ki-67^{+ve} nuclei were counted. Data are presented as the percentage of Ki-67^{+ve} nuclei per crypt.

Whole-mount organoid immunofluorescence images were analysed using Fiji/ImageJ. For each experimental condition, n = 4 organoids were assessed. Within each organoid, at least 20 intestinal epithelial cells (IECs) displaying clear apico-basal orientation were selected from three independent z-sections. Measurements from individual cells were averaged to generate a single mean value per organoid. IEC length was measured using the line tool as the distance from the apical (luminal) surface to the basal region of the crypt, following the longitudinal axis of the cell, while IE width was measured as the lateral distance between opposing cell membranes, perpendicular to the apical-basal axis. All measurements were recorded in micrometres (µm).

Quantification of the histological colitis score (HCS) was performed using the Aperio ImageScope system (v12.4.2.5010), following the method outlined in (47). Briefly, three distinct layers were defined using different colours: total tissue length, areas of inflammation/injury and areas of erosion/ulceration. The “Pen” tool was used to manually outline the tissue, and the length of each category was measured. The sum of the total segment lengths for each region were then computed, and the percentage of injury and ulceration relative to total length was calculated. HCS was determined using the following formula, based on the assumption that complete epithelial loss (i.e. erosion or ulceration) leads to maximal barrier dysfunction and worsened disease outcomes:

$$HCS = \frac{\%inflammation\ or\ injury + 2(\%erosion\ or\ ulceration)}{10}$$

For goblet cell theca area quantification, individual goblet cell theca areas were manually outlined using the “Pen” tool in Aperio ImageScope v12.4.2.5010. The average area per goblet cell was determined by measuring >100 goblet cells per animal.

For quantification of PAS-AB^{+ve} staining in the upper or lower crypts, only fully visible crypts were included in the analysis. PAS-AB^{+ve} stained regions in the top or bottom half of the crypt were manually outlined and measured using the "Pen" tool in Aperio ImageScope.

For quantification of PAS-AB^{+ve} staining in the colon epithelium, analysis was performed using Fiji/ImageJ. The length of the muscularis mucosae was measured using the Line tool to obtain a reference length for normalization. The areas of interest (i.e. distal colon) were isolated by defining a Region of Interest (ROI). Colour deconvolution was applied using the "Colour Deconvolution 2" tool to separate PAS^{+ve}, AB^{+ve}, and haematoxylin staining, allowing mucin staining to be distinguished from background and nuclear staining. PAS^{+ve} and AB^{+ve} channels were then thresholded (Image>Adjust>Threshold (default)) to generate binary images. Watershed separation plugin (Process>Binary>Watershed) was applied to separate the structures. PAS^{+ve} or AB^{+ve} areas were quantified by analysing the structures of interest within the ROI (Analyze>Analyze Particle). The total PAS-AB^{+ve} mucin area was obtained by summing the quantified PAS^{+ve} and AB^{+ve} areas.

For quantification of immunofluorescence staining, Fiji/ImageJ was used. Spatial calibration was first conducted by setting the appropriate scale for each image (Analyse>Set Scale). Quantification of the size of RAB5 vesicles was performed by manually drawing an ROI comprising of more than 5 contiguous intestinal epithelial cells displaying clear apical-basal orientation, while excluding non-specific or luminal debris staining in the organoid lumen, using the polygonal, freehand and line selection tools. The RAB5 channel was then thresholded to create a binary image containing the

structures of interest (Image>Adjust>Threshold (default)) followed by the application of the watershed separation plugin (Process>Binary>Watershed) to separate the structures (referred to herein as particles). The particles within the ROI were then analysed (Analyze>Analyze Particle) to obtain the average size of particle.

Quantification of apical, lateral, basal and cytoplasmic fluorescence signals on whole-mount fluorescence images was conducted by manually drawing ROIs around the relevant structures using the polygonal, freehand and line selection tools and measured using the 'Measure' (Analyse>Measure) function. A minimum of 3 ROIs (comprising of >5 cells one after another) per stack, and at least 3 z-sections with clear apical-to-basal orientation, were analysed per organoid.

Co-localisation analysis was performed using the Just Another Colocalisation Plugin (JaCoP; Bioimaging and Optics Platform, BIOP). Otsu thresholding was applied to define object boundaries.

Statistics and reproducibility

Numerical source data for all graphs are provided in Materials and Methods and Figure legends. Statistical tests were performed using GraphPad Prism 8 Software (GraphPad, San Diego, California, USA) *via* Student's unpaired t-tests when comparing between two groups. One-way ANOVA or two-way ANOVA (with Tukey post-hoc comparisons) was used for multiple comparisons. Each mouse was assessed as an individual sample. All data were obtained by performing at least $n > 2$ independent experiments with representative data shown and expressed as the mean ± standard error of the mean

(S.E.M). *P values* < 0.05 were considered as statistically significant. Significance levels were split further as follows: $^{**}P < 0.01$, $^{***}P < 0.001$, $^{****}P < 0.0001$. Variance between groups was visually assessed and found to be comparable, and statistical tests appropriate for the data structure were applied, including unpaired Student's t-tests and one way-ANOVA, which are robust to modest variance differences. Sample sizes were based on those commonly used in prior intestinal BECLIN1 studies and DSS-colitis models. Although no formal prospective power calculation was performed, group sizes were sufficient to detect biologically meaningful differences and are reported for each experiment in the Figure Legends and Methods. No animals or samples were excluded from the study except in cases of technical compromise (e.g. damaged or poorly oriented crypts).

SUPPLEMENTARY FIGURE LEGENDS

Supplementary Figure 1. Monoallelic *Becn1* deletion in mice results in lower BECLIN1 protein levels but autophagy was not significantly impacted. (A) Representative PCR genotyping of IECs isolated from different sections of the gastrointestinal tract of *n* = 3 *Becn1*$^{+/+}$;*Vil1-CreERT2*$^{Cre/+}$ and *Becn1*$^{fl/+}$;*Vil1-CreERT2*$^{Cre/+}$ mice two weeks post-Tamoxifen treatment. Numbers (1-6) correspond to individual animals. Untreated IEC controls (*+/+, fl/+* and *fl/fl*) were isolated from the duodenum of *Becn1*$^{+/+}$*;, Becn1*$^{fl/+}$*;,* and *Becn1*$^{fl/fl}$;*Vil1-CreERT2*$^{Cre/+}$ mice without Tamoxifen treatment. Monoallelic *Becn1* deletion (Pos *fl/+*) is evidenced by the presence of a 398 bp band corresponding to the 'wild-type (WT)' allele, the loss or significant reduction of the 510 bp 'Floxed' band, and the emergence of a 721 bp band corresponding to the deleted allele

(KO). **(B)** Western blots used for quantitation of relative BECLIN1 levels shown in Figure 1A of main manuscript. Samples represent *n* = 6 *Becn1*$^{IEC+/+}$ and *Becn1*$^{IEC+/-}$ mice and *n* = 3 *Becn1*$^{IEC-/-}$ mice. **(C)** Quantitation of band intensities for Western blots shown in Figure 1E of the main manuscript. For P62 (left panel), band volumes were normalised to the β-actin band in the same lane. LC3B-I:LC3B-II ratios (middle panel) represent the ratio of each absolute band volume and total LC3B (right panel) is the sum of LC3B-I+LC3B-II band volumes normalised to the β-actin band in the same lane. SI: small intestine. Duo: Duodenum. Jej: jejunum. Ile: ileum. Col: colon. BP: base pairs. Pos: positive control. Neg: negative control.

Supplementary Figure 2. Monoallelic *Becn1* deletion in mice results in shortened small intestines and reduced colonic crypt length even when aged up to one month. **(A)** Body weight of mice over time, normalised to day 0. Data represent *n* = 9 biologically independent mice of each genotype from *n* = 3 independent experiments. **(B)** Distal colon crypt width of *Becn1*$^{IEC+/+}$ and *Becn1*$^{IEC+/-}$ mice at days 7 and 35 post-tamoxifen administration. Data represent *n* = 4 to 8 animals per genotype from *n* = 3 independent experiments. **(C)** Representative images of intestinal tracts of *Becn1*$^{IEC+/+}$ and *Becn1*$^{IEC+/-}$ mice along with the measurements of intestinal length at Day 35 post Tamoxifen administration. Data represent *n* = 3 to 4 animals per genotype from *n* = 3 independent experiments. **(D)** H&E-stained FFPE sections of *Becn1*$^{IEC+/+}$ and *Becn1*$^{IEC+/-}$ intestinal tracts at 35 days post-tamoxifen administration. Scale bars = 200 µm. Data represent *n* = 6 mice from *n* = 3 independent experiments. Graphs show the mean ± S.E.M. Statistical

significance was determined using unpaired (Student's) t-test in (B) and (C), and changes in body weight in (A) were determined using two-way ANOVA with Tukey's post-hoc test.

Supplementary Figure 3. BECLIN1 reduction in intestinal organoids does not inhibit basal autophagic function. (A) Representative Western blot detecting BECLIN1 levels in *Becn1*$^{IEC+/+}$, *Becn1*$^{IEC-/-}$ and *Becn1*$^{IEC+/-}$ organoids at day 7 post-4HT treatment. **(B)** Western blot assessment of basal autophagy in *Becn1*$^{IEC+/+}$, *Becn1*$^{IEC+/-}$ and *Becn1*$^{IEC-/-}$ organoids, analysing total levels of P62, LC3B-I and LC3B-II, with GAPDH serving as a loading control. Images are representative from *n* = 3 independent experiments with *n* = 3 different biological replicates.

Supplementary Figure 4. Altered endocytic trafficking and epithelial cell morphology in intestinal organoids with reduced BECLIN1. (A) Representative images of whole-mount immunostained intestinal organoids from *Becn1*$^{IEC+/+}$, *Becn1*$^{IEC-/-}$ and *Becn1*$^{IEC+/-}$ mice showing RAB5 and E-CADHERIN staining, analysed using BioP-JACoP colocalisation with Otsu thresholding. Colocalised regions between RAB5 and E-CADHERIN are visualised in the MERGE (white) channel. Images are representative from *n* = 3 independent experiments with *n* = 4 biologically independent organoids per genotype. Scale bar = 5 μm. **(B)** Representative whole-mount immunofluorescence images of intestinal organoids used for epithelial cell morphometric analysis. Images show representative organoids from *n* = 4 biologically independent organoids per genotype, illustrating how intestinal epithelial cell length and width were quantified. Scale bar = 10 μm.

Supplementary Figure 5. Quantification strategy and representative images for PAS-AB mucin analysis in the colon. (A) Representative PAS-Alcian (PAS-AB)-stained sections of the distal colons from *n* = 3 biologically independent animals per genotype, used for quantification shown in Figures 4B to D. **(B)** Representative PAS-AB-stained distal colonic epithelium illustrating the boundary used to define upper and lower crypt regions for all mucin quantifications, as indicated by the dashed line. Black scale bar = 100 µm. Red scale bar = 70 µm.

Supplementary Figure 6. Baseline characteristics and intestinal length measurements of *Becn1*$^{IEC+/+}$ and *Becn1*$^{IEC+/-}$ mice following DSS or control treatment. (A) Starting weights of mice used in the experiments, measured at day zero prior to Tamoxifen injections. **(B)** The average amount of daily 2% DSS water intake, obtained by calculating the difference in weights of drinking water bottles at the start and end of experiment and dividing by the number of days the animals received treatment. **(C)** Small intestinal lengths of *Becn1*$^{IEC+/+}$ and *Becn1*$^{IEC+/-}$ mice who received normal drinking water (untreated) or 2% DSS drinking water (2% DSS) at endpoint. **(D)** Number and **(E)** size of lymphoid follicles in the colon of *Becn1*$^{IEC+/+}$ and *Becn1*$^{IEC+/-}$ mice receiving normal drinking water or 2% DSS drinking water, calculated using H&E stained FFPE colon sections. In all graphs, data are representative of *n* = 9 biologically independent mice per genotype and treatment group from *n* = 3 independent experiments. Graphs indicate the ± S.E.M. Statistical significance was determined using ordinary one-way ANOVA except in (B) where unpaired (Student's) t-test was used.

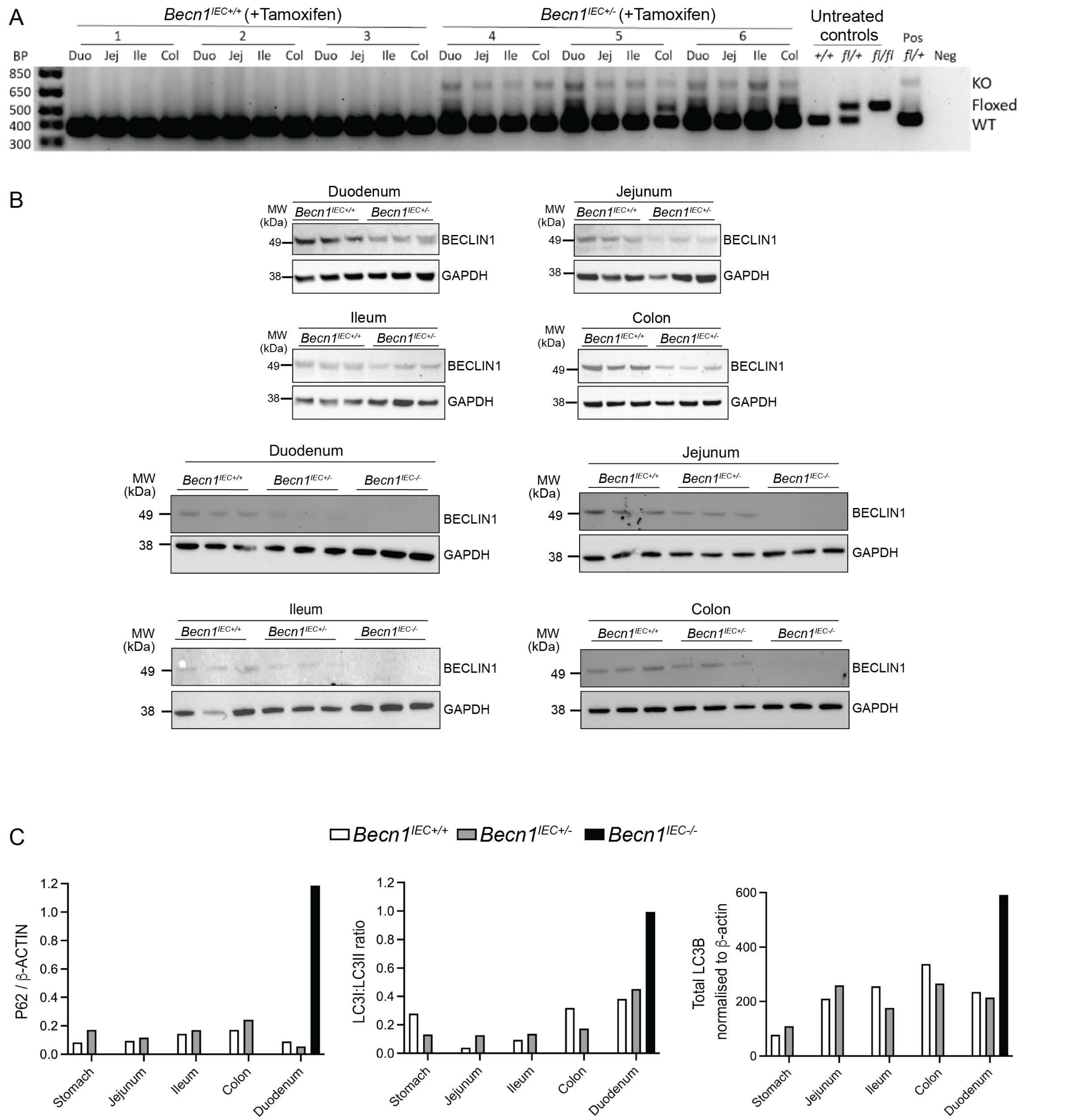
A
Becn1IEC+/+ (+Tamoxifen)
Becn1IEC+/- (+Tamoxifen)
1
2
3
4
5
6
Untreated controls
BP
850
650
500
400
300
Duo Jej Ile Col
+/+ fl/+ fl/fl
Pos fl/+
Neg
KO
Floxed
WT
B
Duodenum
Jejunum
Ileum
Colon
MW (kDa)
Becn1IEC+/+
Becn1IEC+/-
Becn1IEC-/-
49
38
BECLIN1
GAPDH
C
Becn1IEC+/+ Becn1IEC+/- Becn1IEC-/-
P62 / β-ACTIN
LC3I:LC3II ratio
Total LC3B normalised to β-actin
0.0
0.2
0.4
0.6
0.8
1.0
1.2
0
200
400
600
Stomach
Jejunum
Ileum
Colon
Duodenum

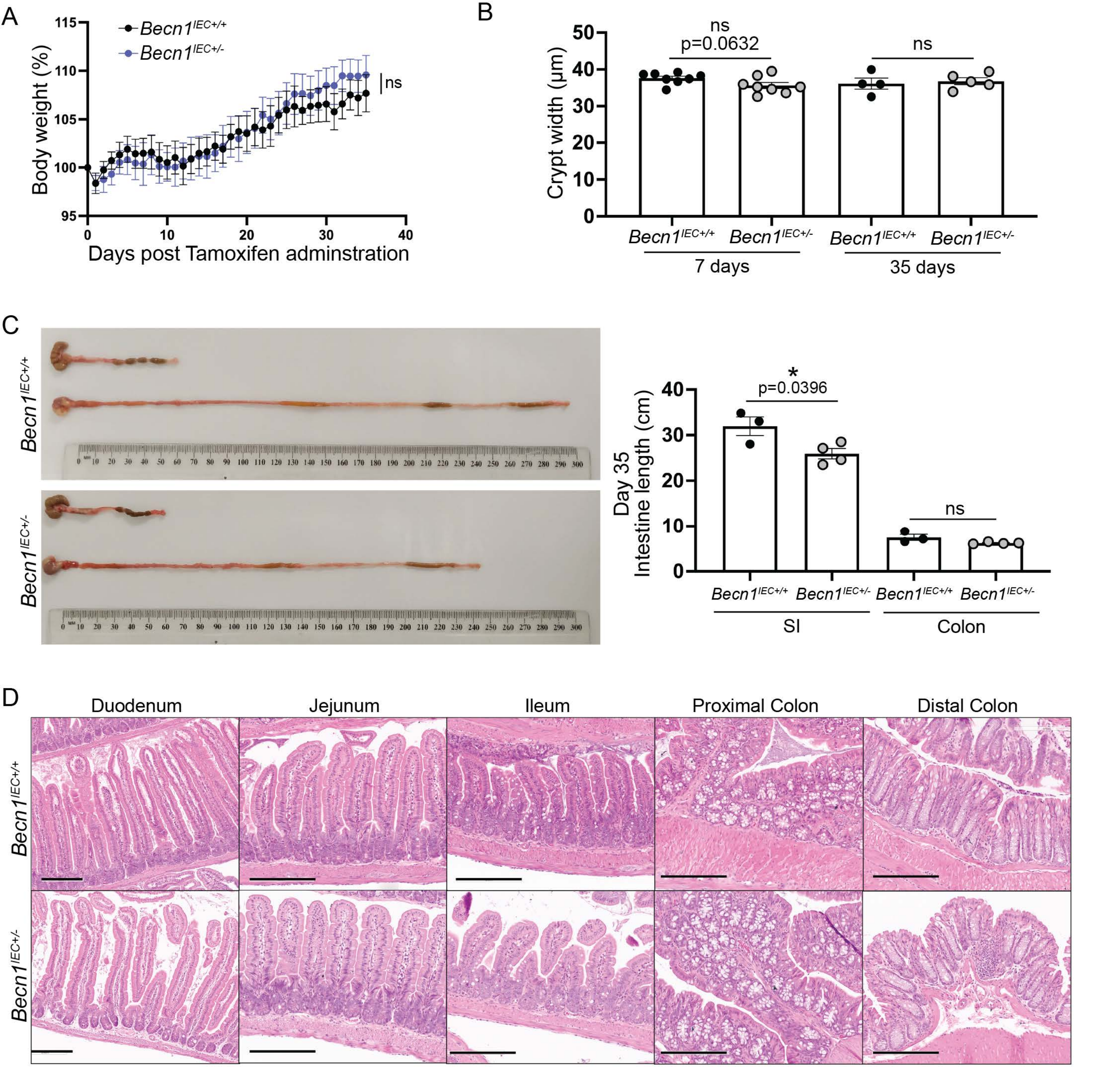

A
Becn1^IEC+/+
Becn1^IEC+/-
Body weight (%)
Days post Tamoxifen adminstration
ns
B
Crypt width (μm)
ns
p=0.0632
ns
Becn1^IEC+/+
Becn1^IEC+/-
7 days
35 days
C
Becn1^IEC+/+
Becn1^IEC+/-
Day 35
Intestine length (cm)
*
p=0.0396
ns
SI
Colon
D
Duodenum
Jejunum
Ileum
Proximal Colon
Distal Colon

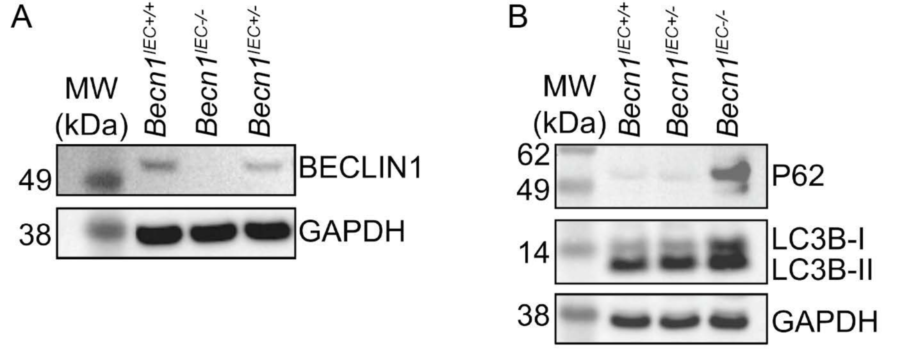
A
MW (kDa)
Becn1 IEC+/+
Becn1 IEC-/-
Becn1 IEC+/-
49
BECLIN1
38
GAPDH
B
MW (kDa)
Becn1 IEC+/+
Becn1 IEC+/-
Becn1 IEC-/-
62
49
P62
14
LC3B-I
LC3B-II
38
GAPDH

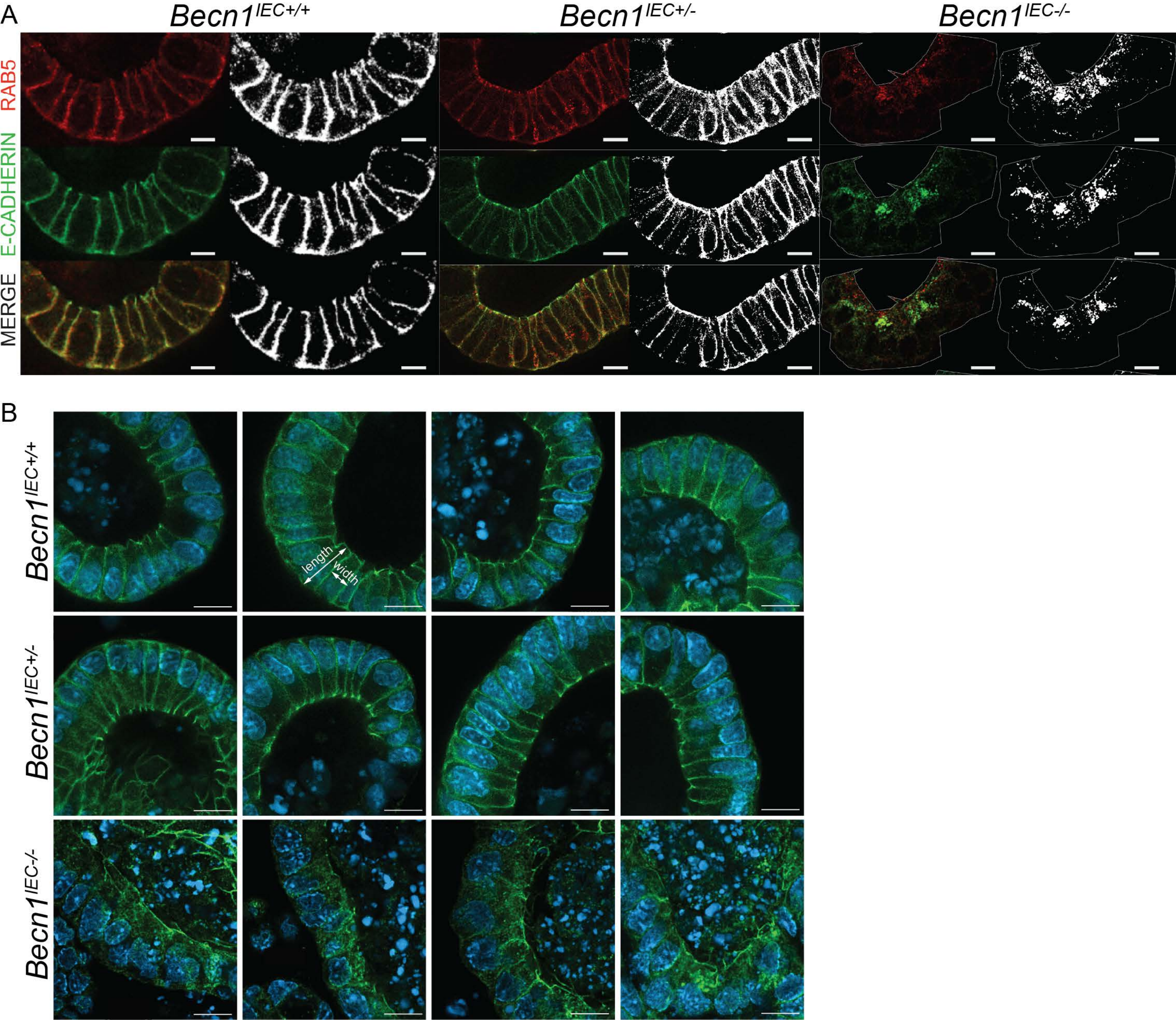
A
Becn1IEC+/+
Becn1IEC+/-
Becn1IEC-/-
RAB5
E-CADHERIN
MERGE
B
Becn1IEC+/+
Becn1IEC+/-
Becn1IEC-/-
length
width

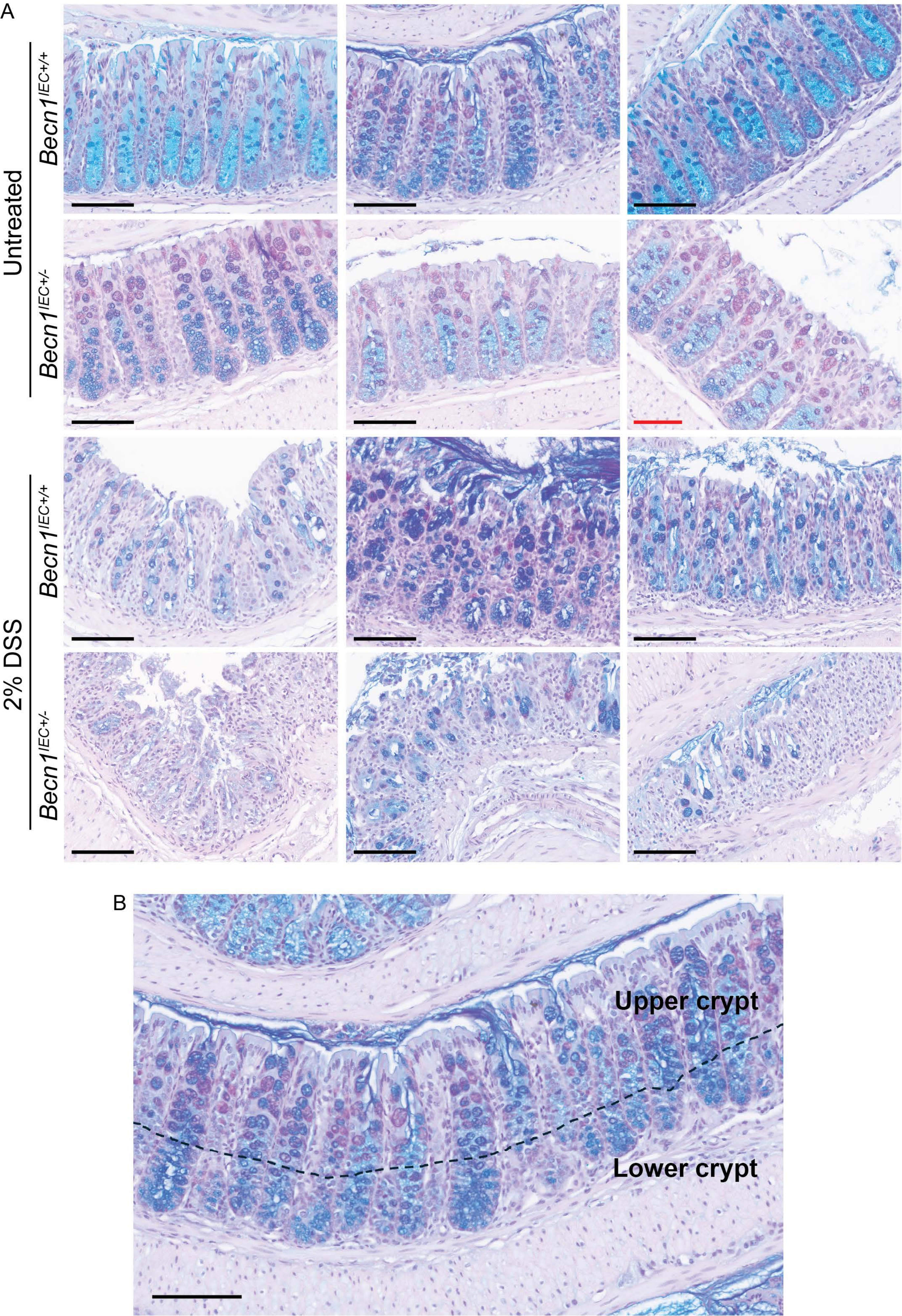

A
Untreated
$Becn1^{IEC+/+}$
$Becn1^{IEC+/-}$
2% DSS
$Becn1^{IEC+/+}$
$Becn1^{IEC+/-}$
B
Upper crypt
Lower crypt

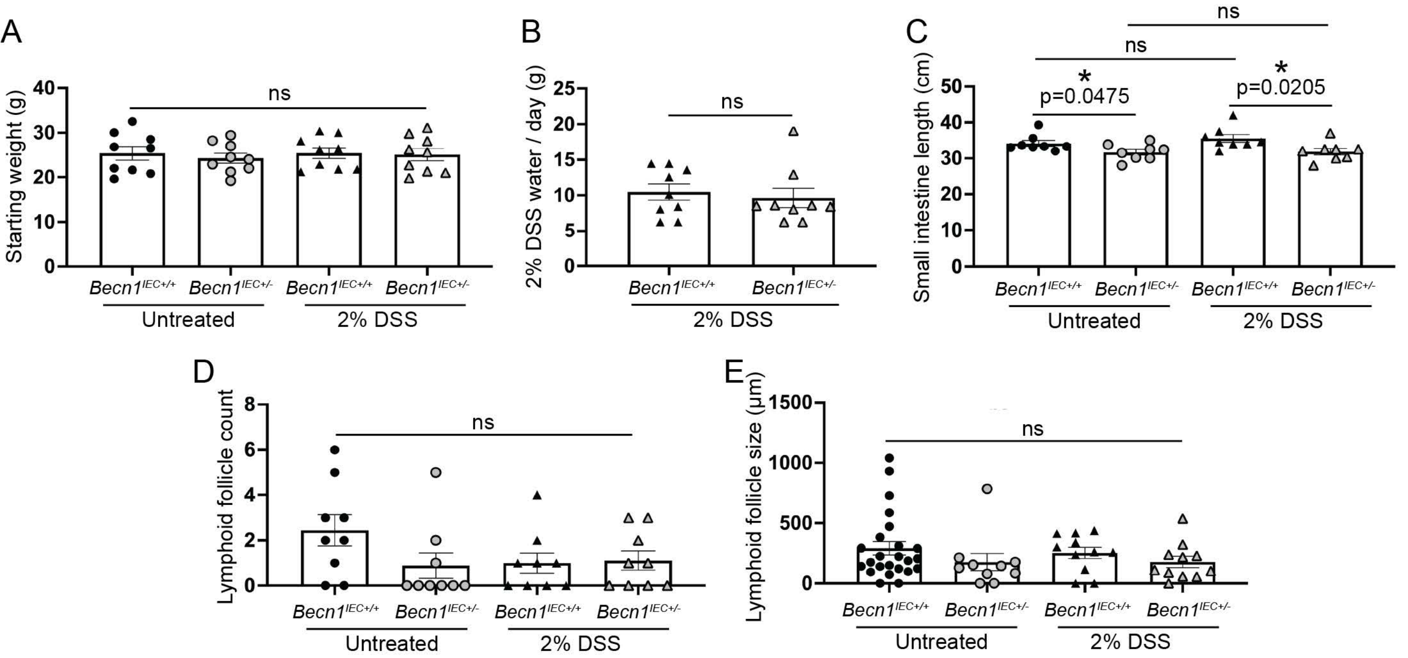
A
Starting weight (g)
ns
Becn1IEC+/+
Becn1IEC+/-
Untreated
2% DSS
B
2% DSS water / day (g)
C
Small intestine length (cm)
p=0.0475
p=0.0205
D
Lymphoid follicle count
E
Lymphoid follicle size (µm)
