# Supplementary material for: Impact of BECLIN1 haploinsufficiency on goblet cell function and susceptibility to colitis
Source: Cell Death Dis. 2026 Jun 17;17(1):637. doi: 10.1038/s41419-026-08984-8 (PMC13365605; doi:10.1038/s41419-026-08984-8)

Figure 1A and Supplementary Figure 1C

Duodenum

Anti-BECLIN1

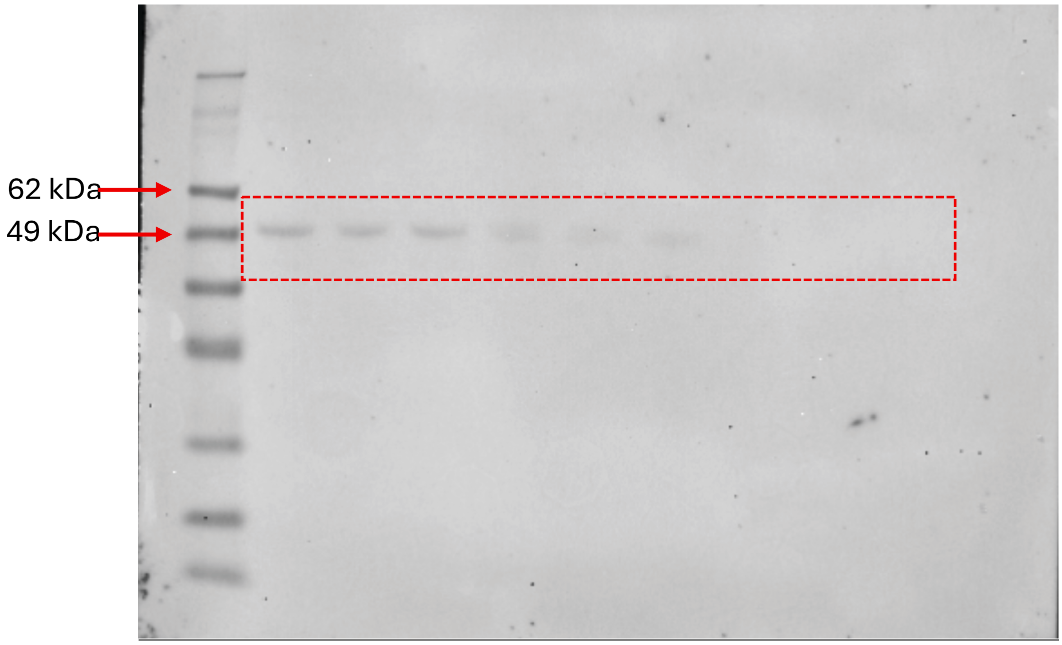

Anti-GAPDH

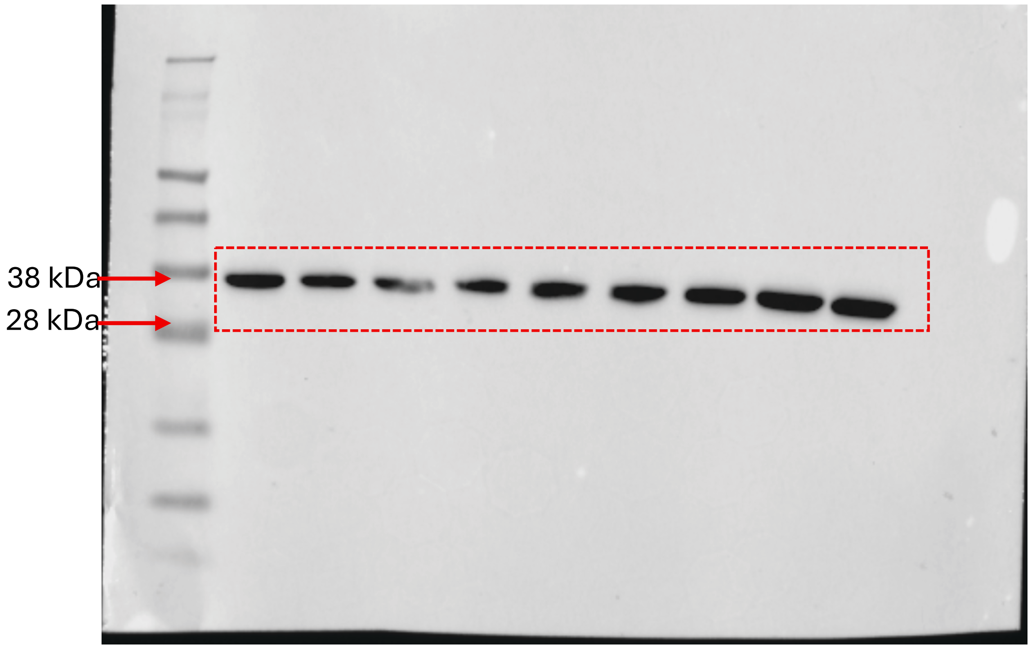

Jejunum

Anti-BECLIN1

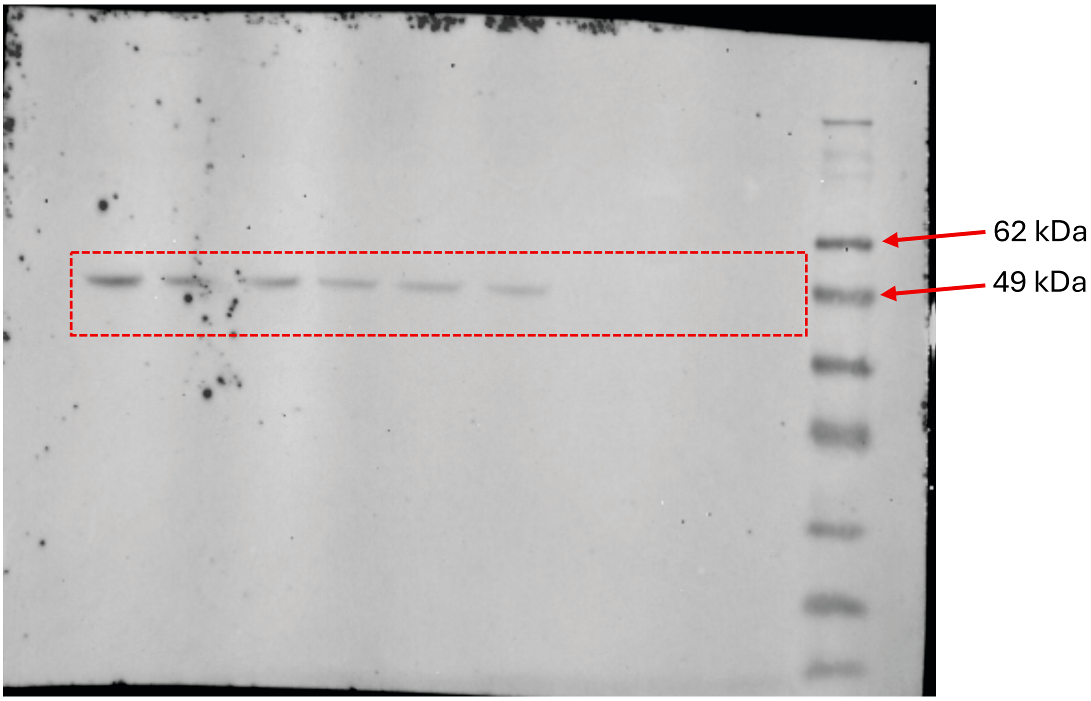

Anti-GAPDH

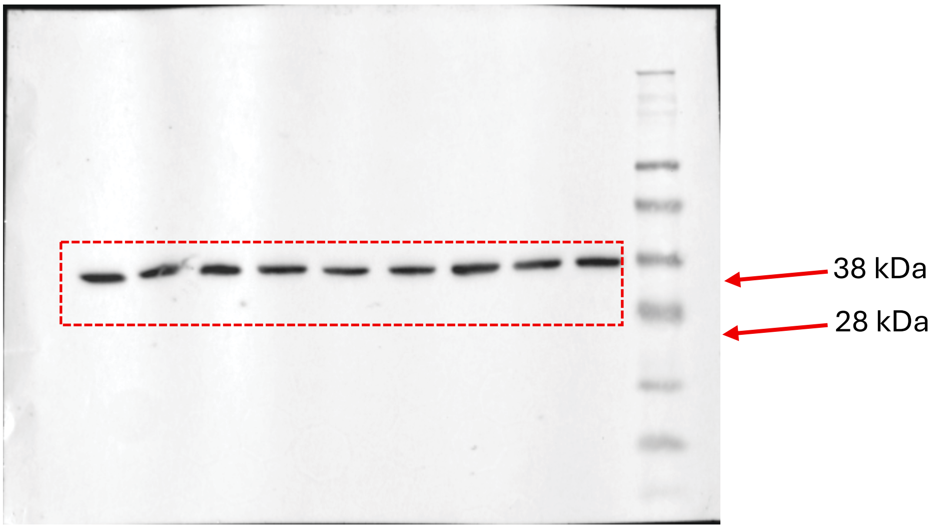

Ileum

Anti-BECLIN1

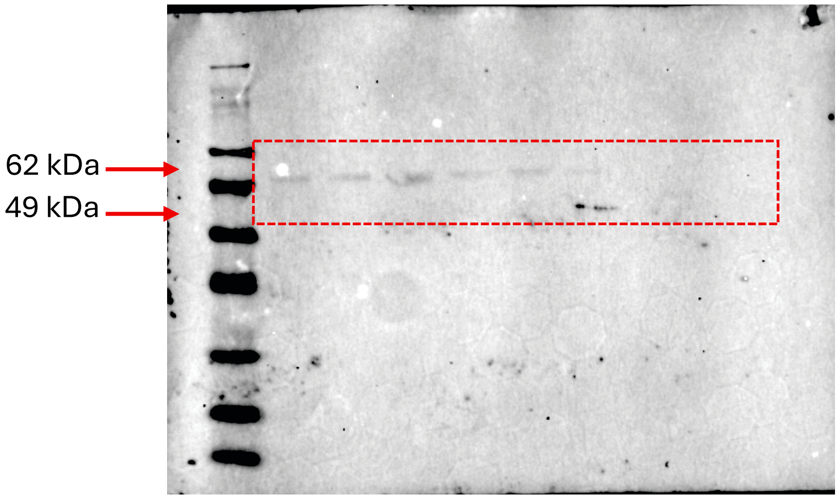

Anti-GAPDH

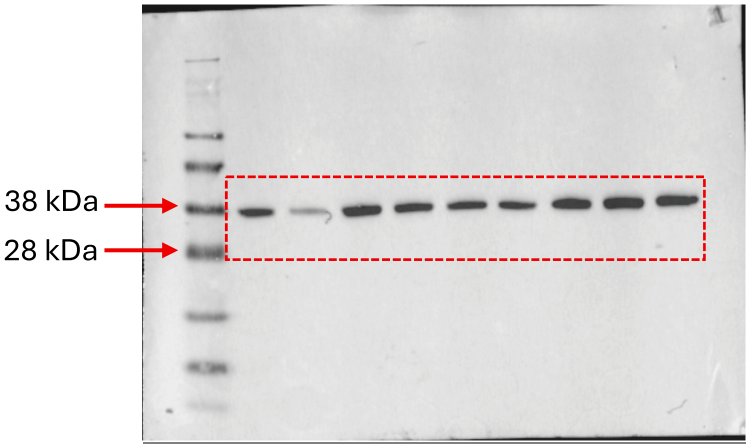

Colon

Anti-BECLIN1

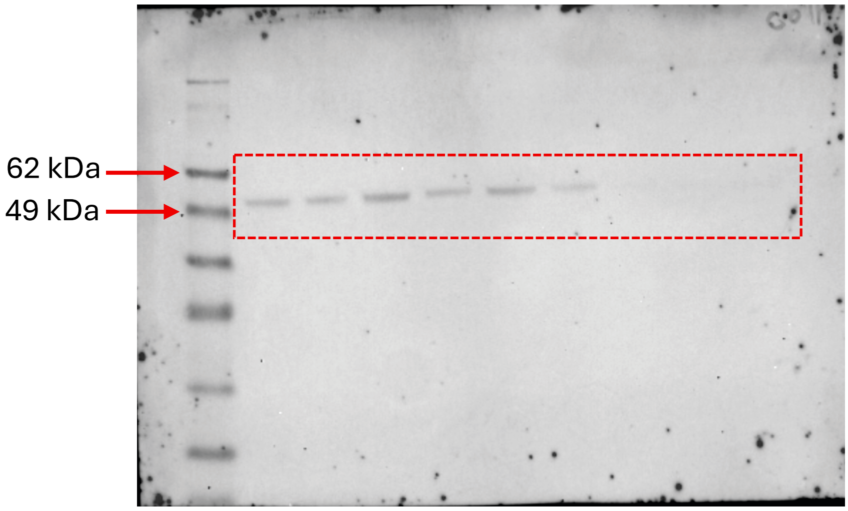

Anti-GAPDH

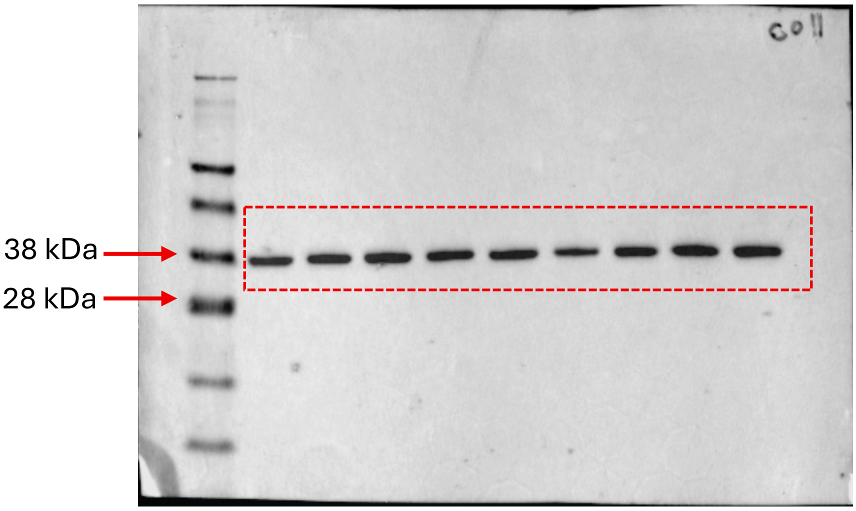

Supplementary Figure 1C

Duodenum

Anti-BECLIN1

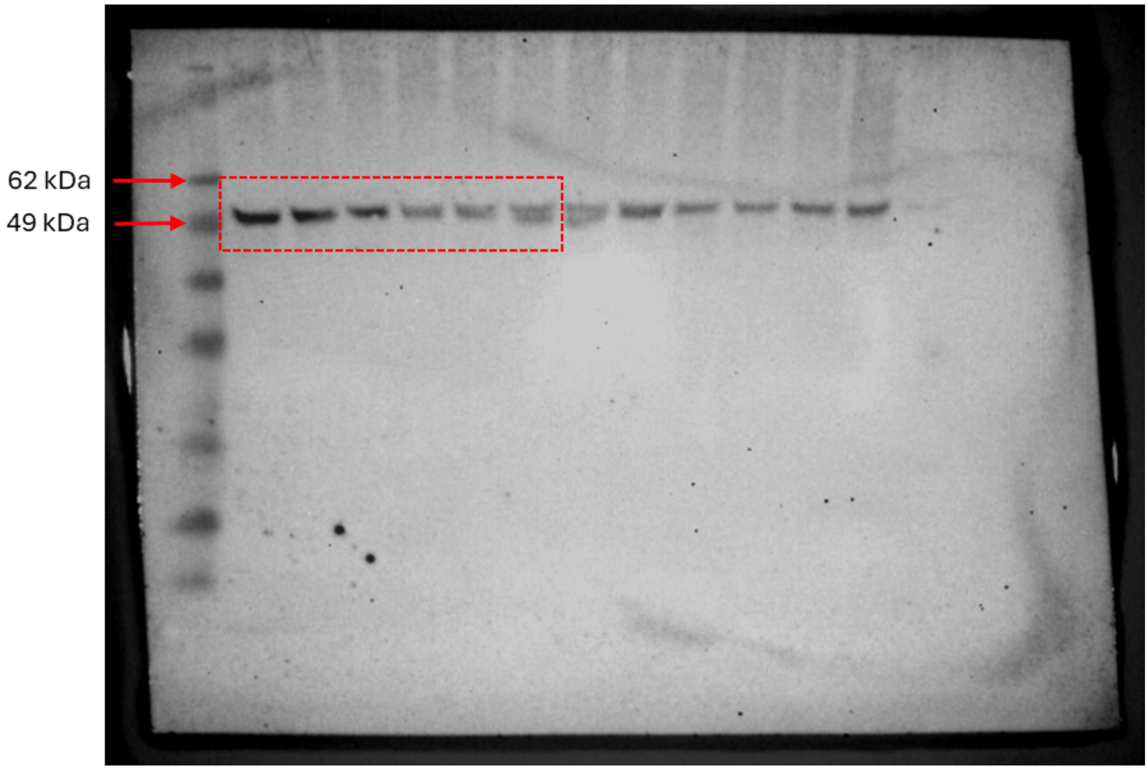

Anti-GAPDH

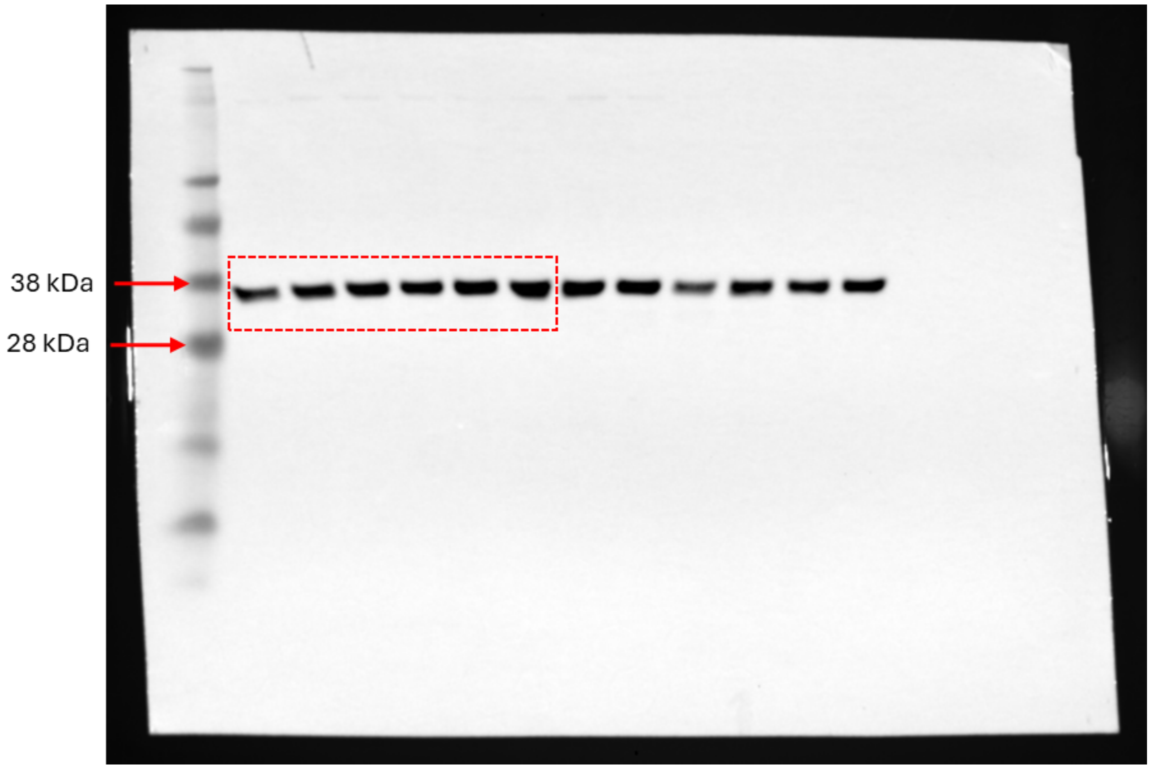

Jejunum

Anti-BECLIN1

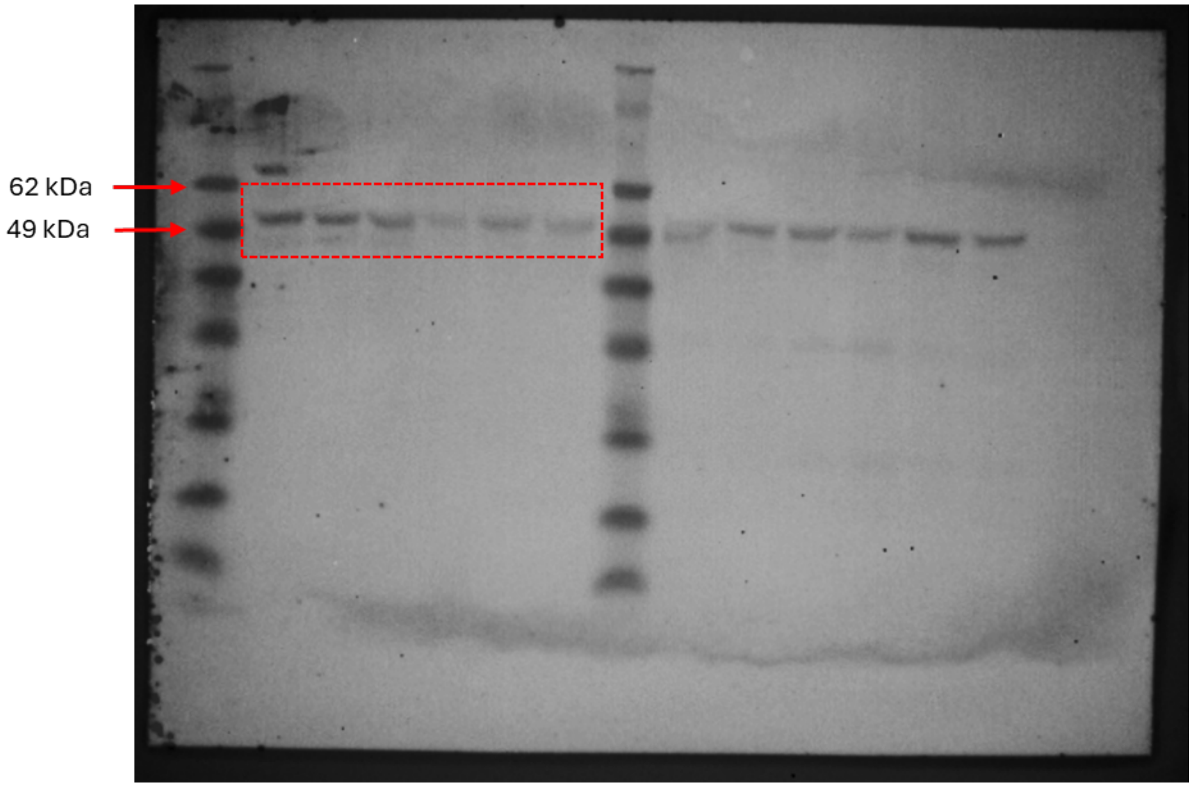

Anti-GAPDH

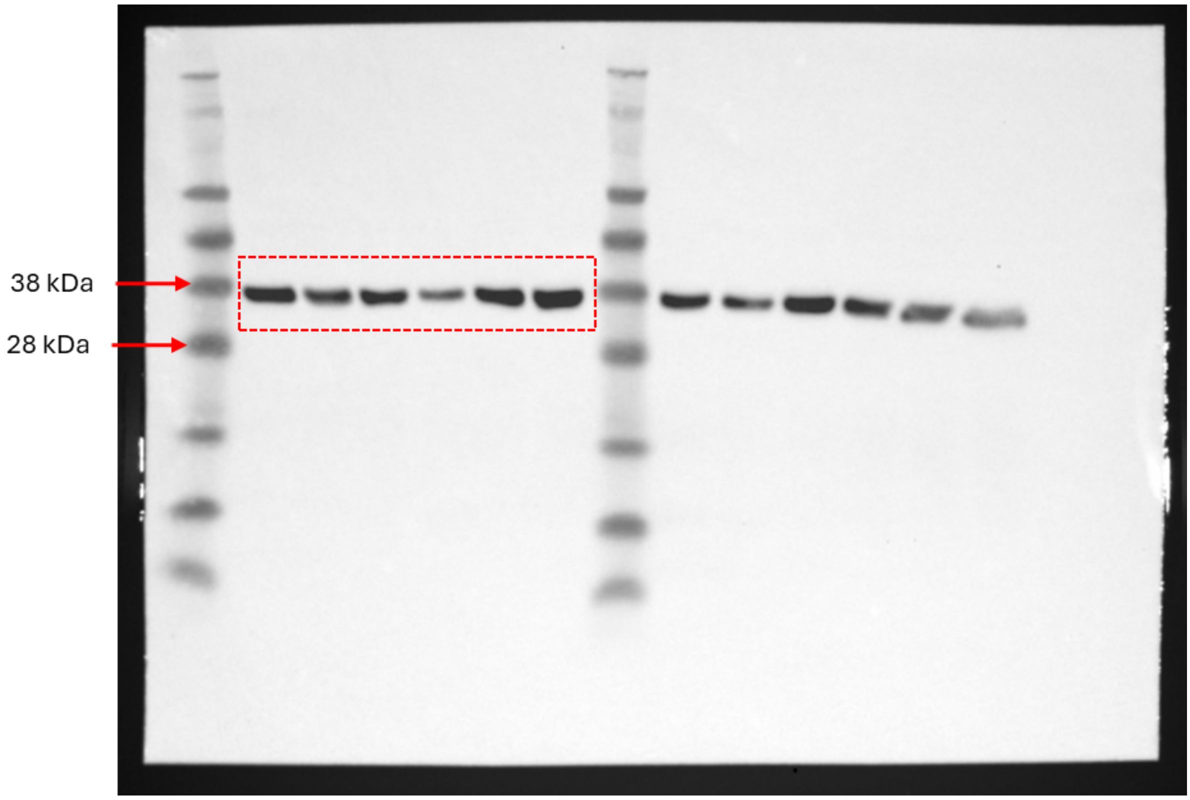

Ileum

Anti-BECLIN1

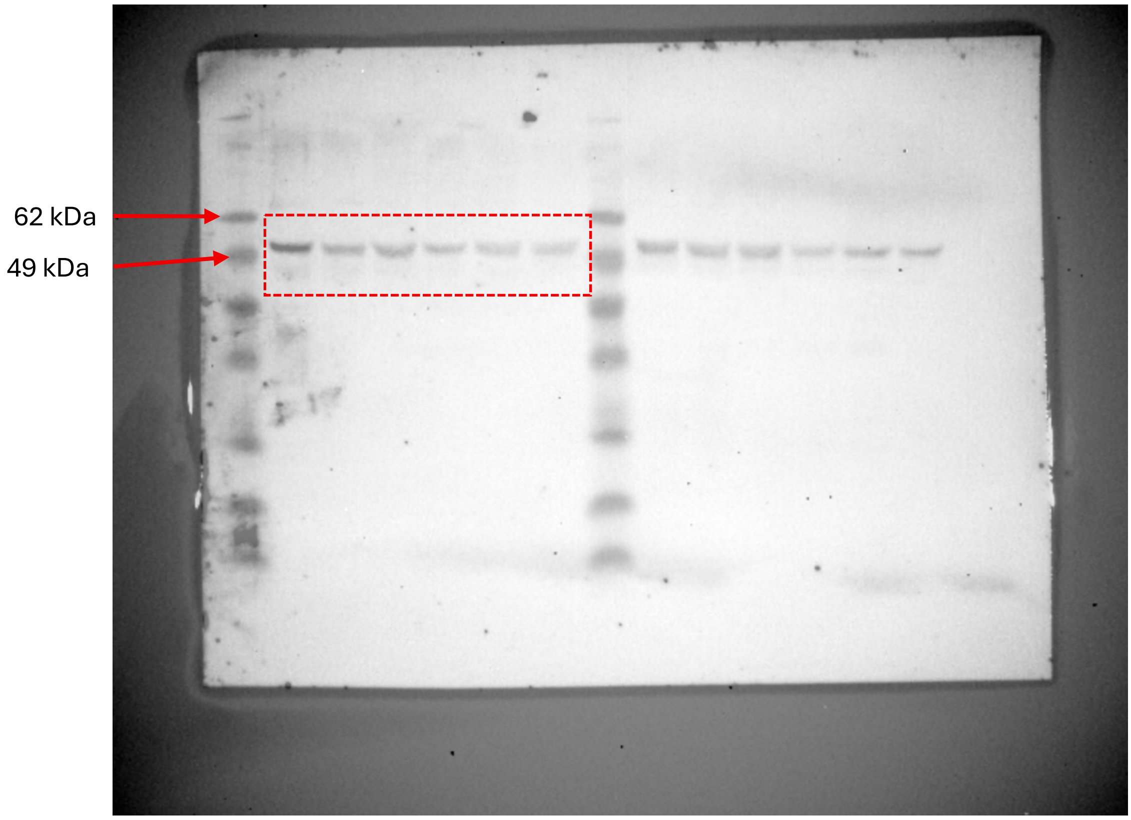

Anti-GAPDH

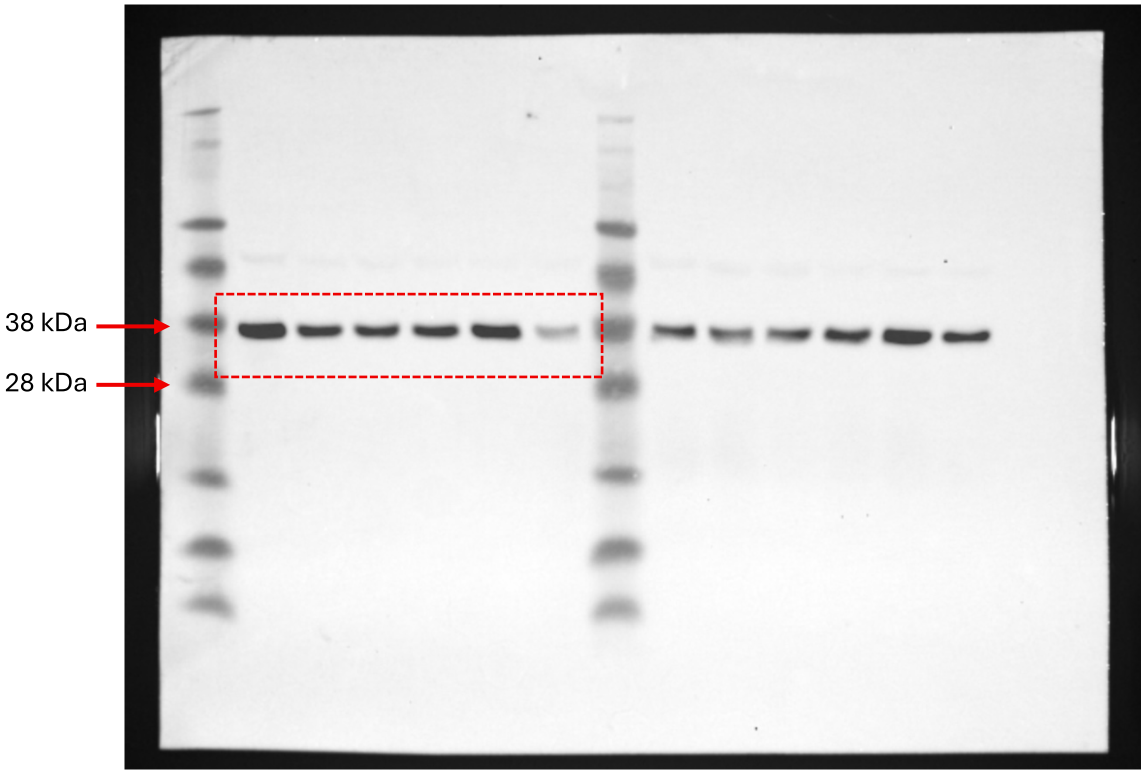

Colon

<u>Anti-BECLIN1</u>

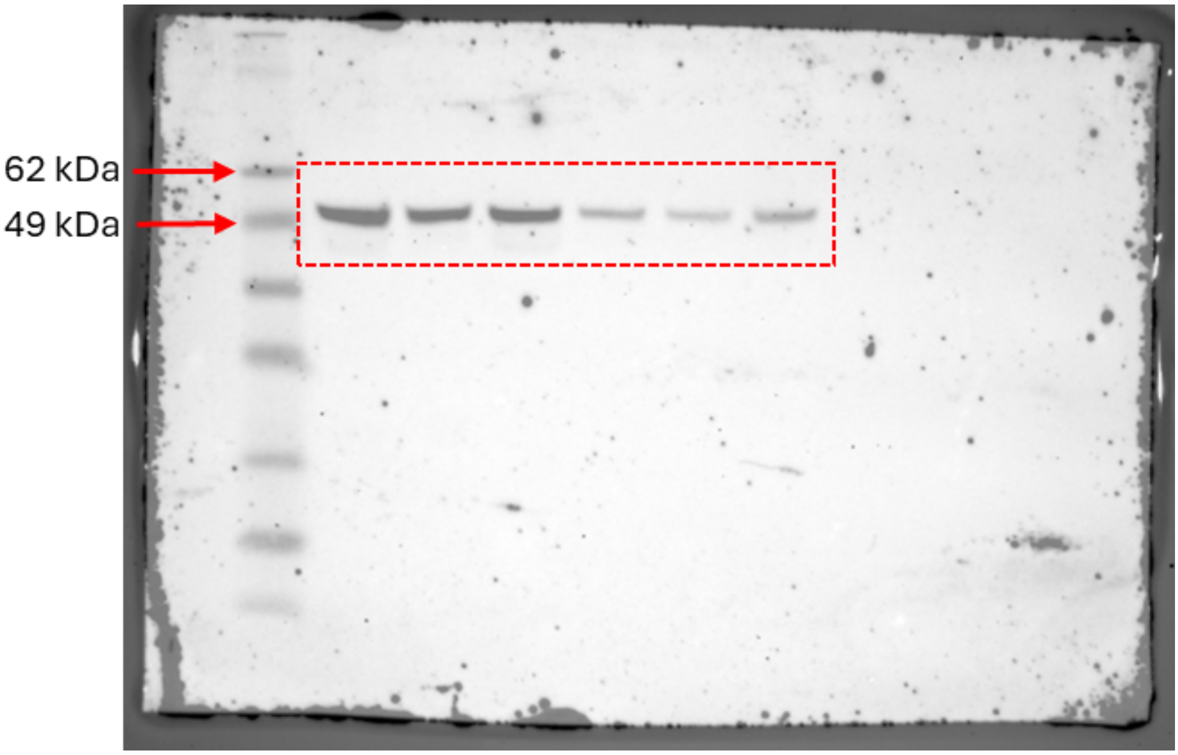

<u>Anti-GAPDH</u>

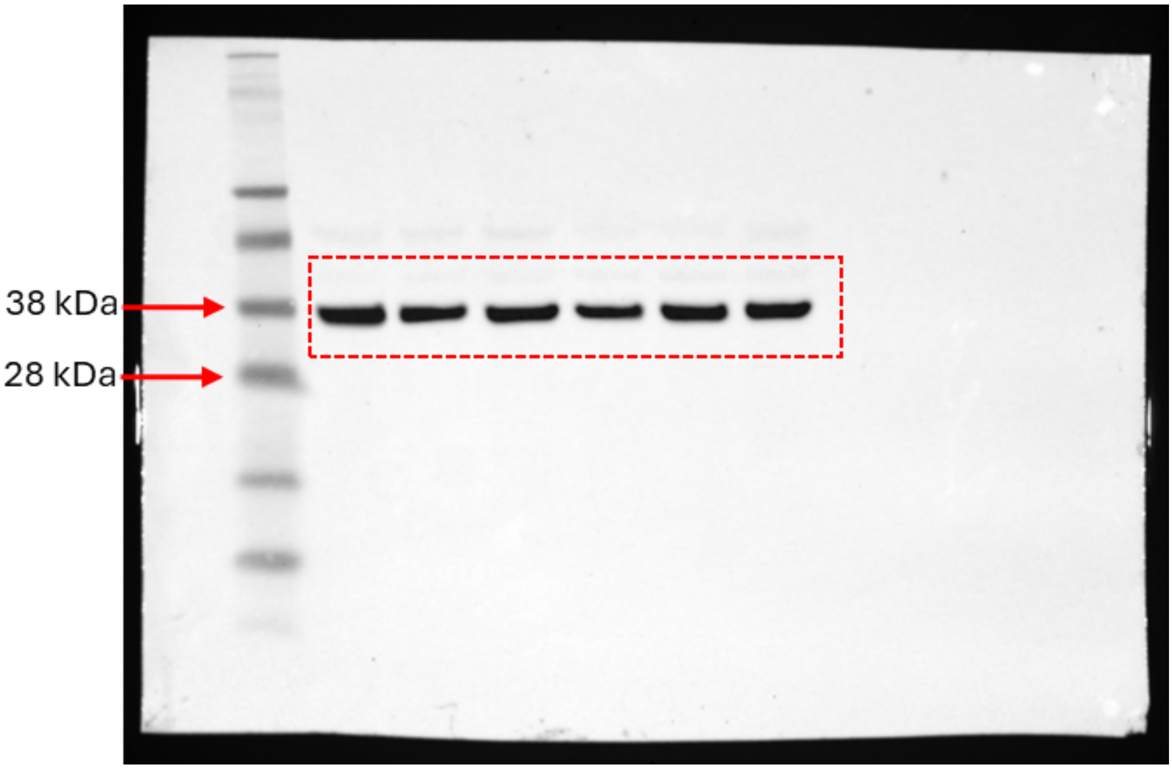

Figure 1E

Anti-P62

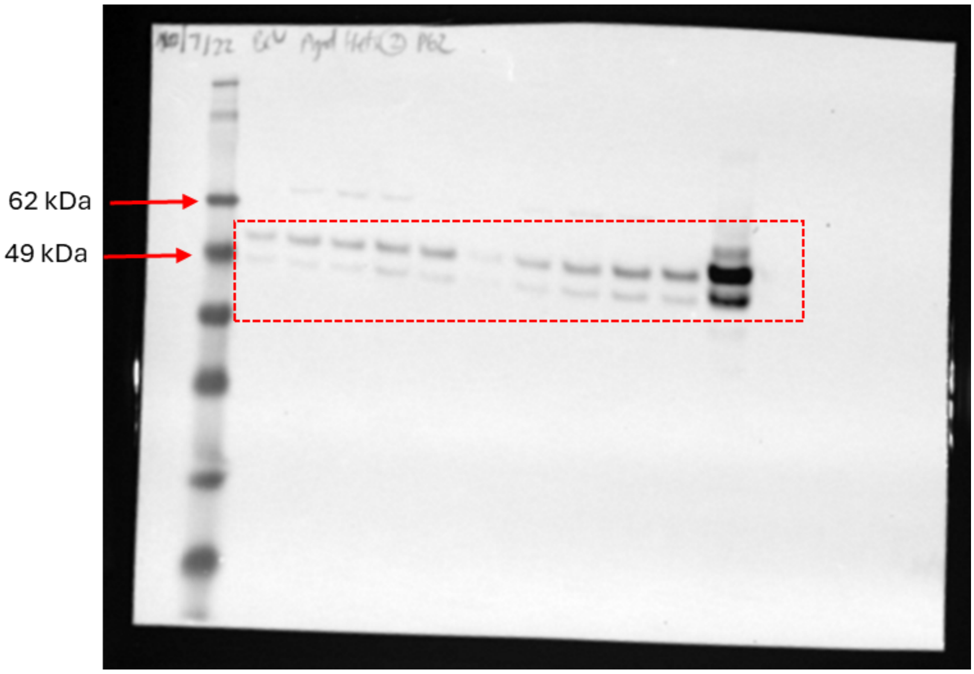

Anti-LC3B

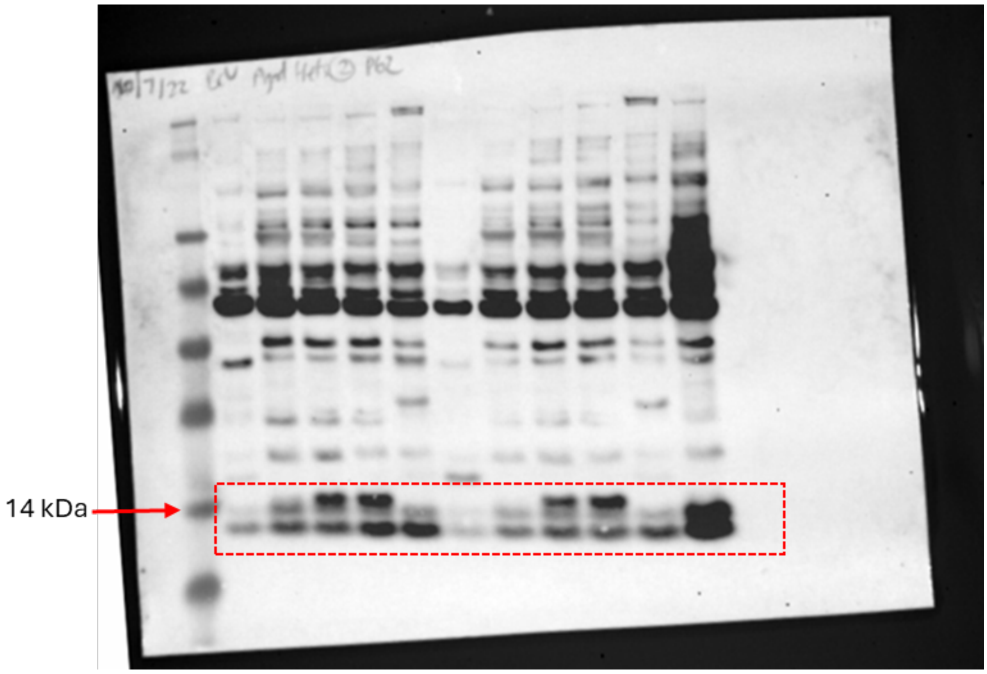

Anti-β-ACTIN

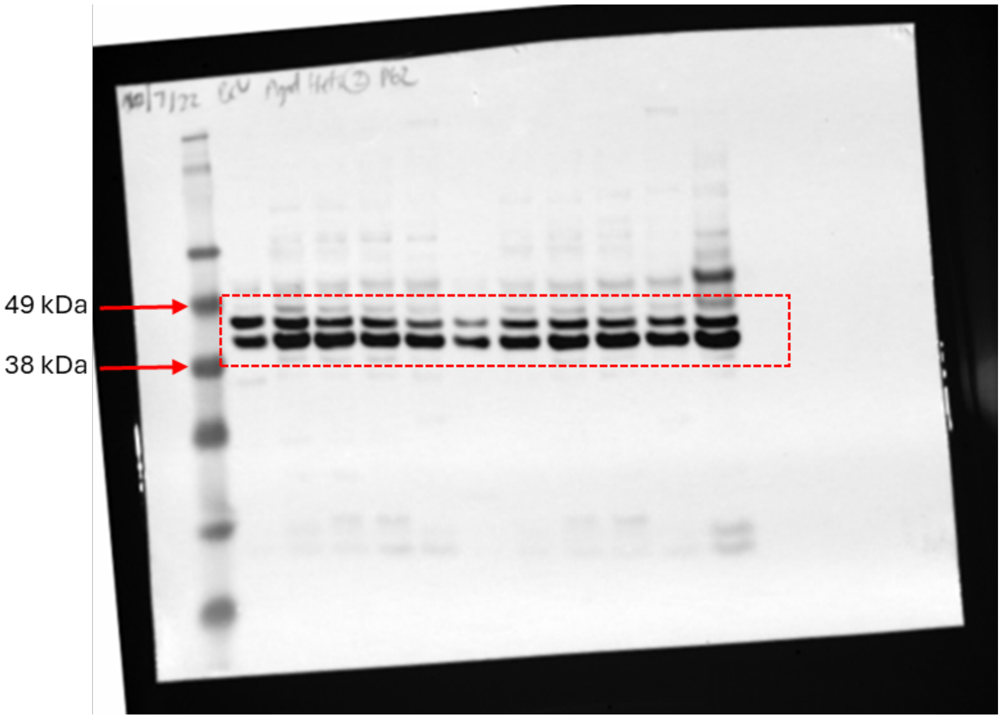

Supplementary Figure 3A

Anti-BECLIN1

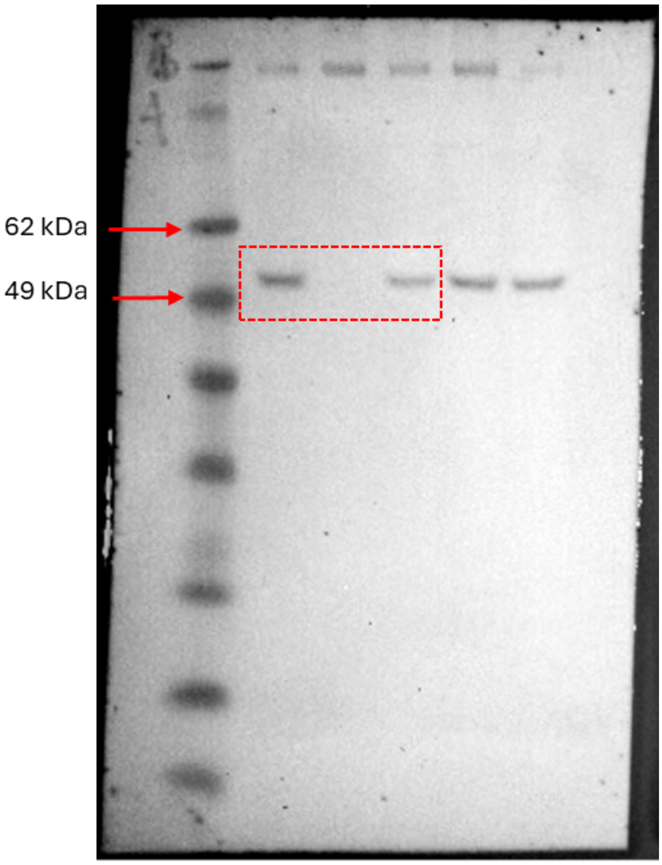

Anti-GAPDH

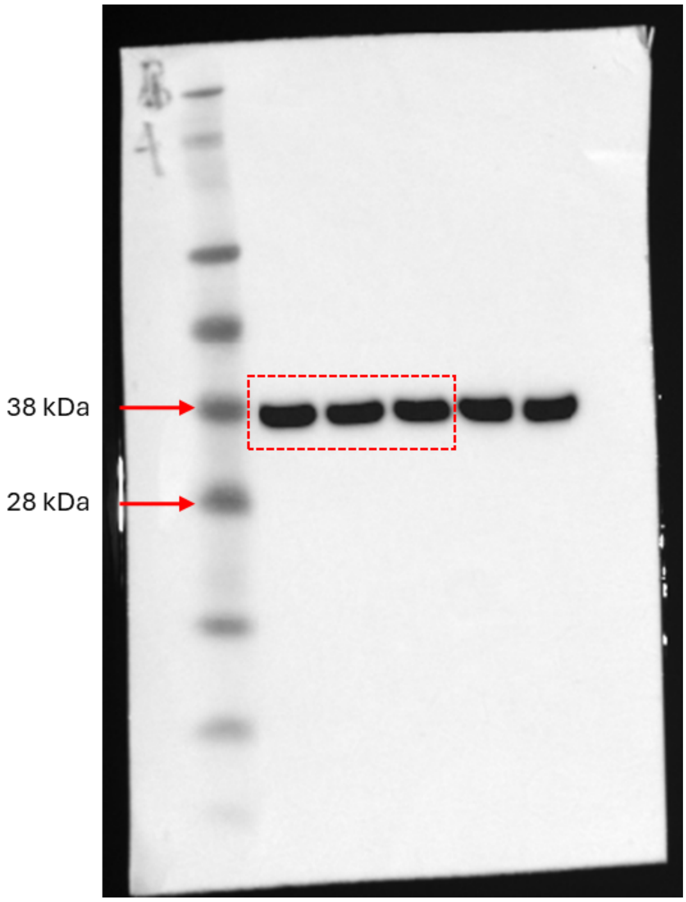

Supplementary Figure 3B

Anti-P62

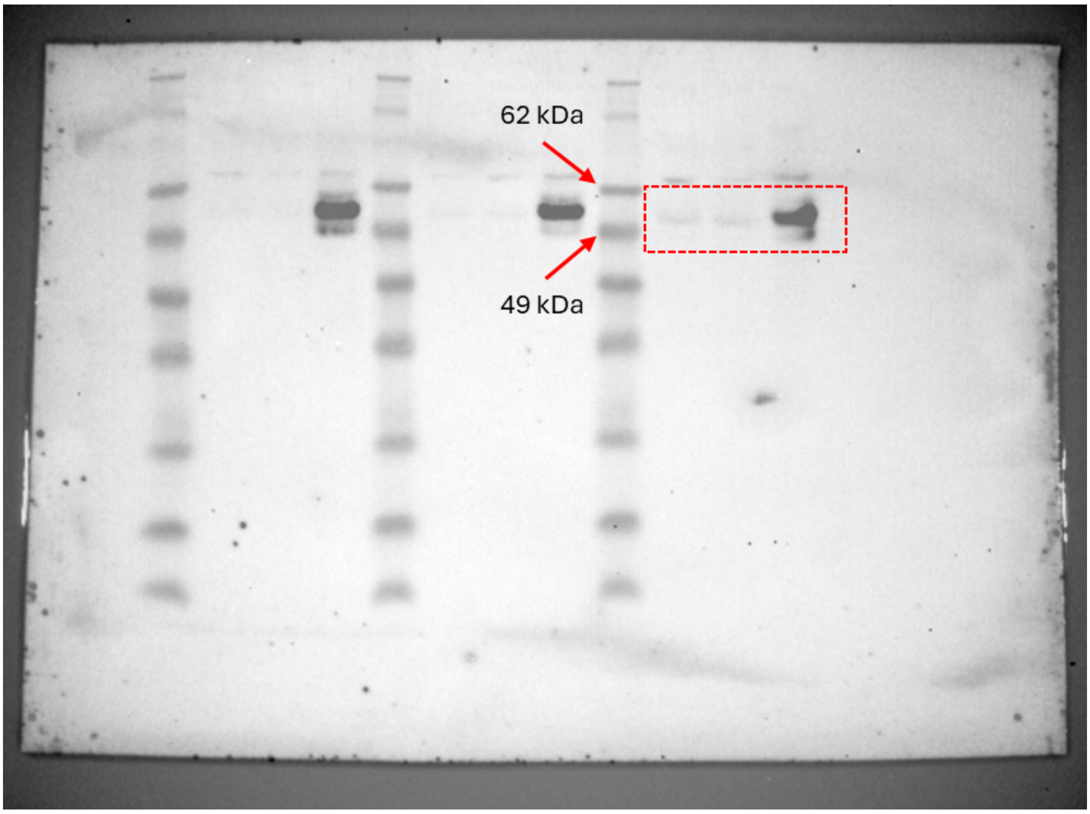

Anti-LC3B

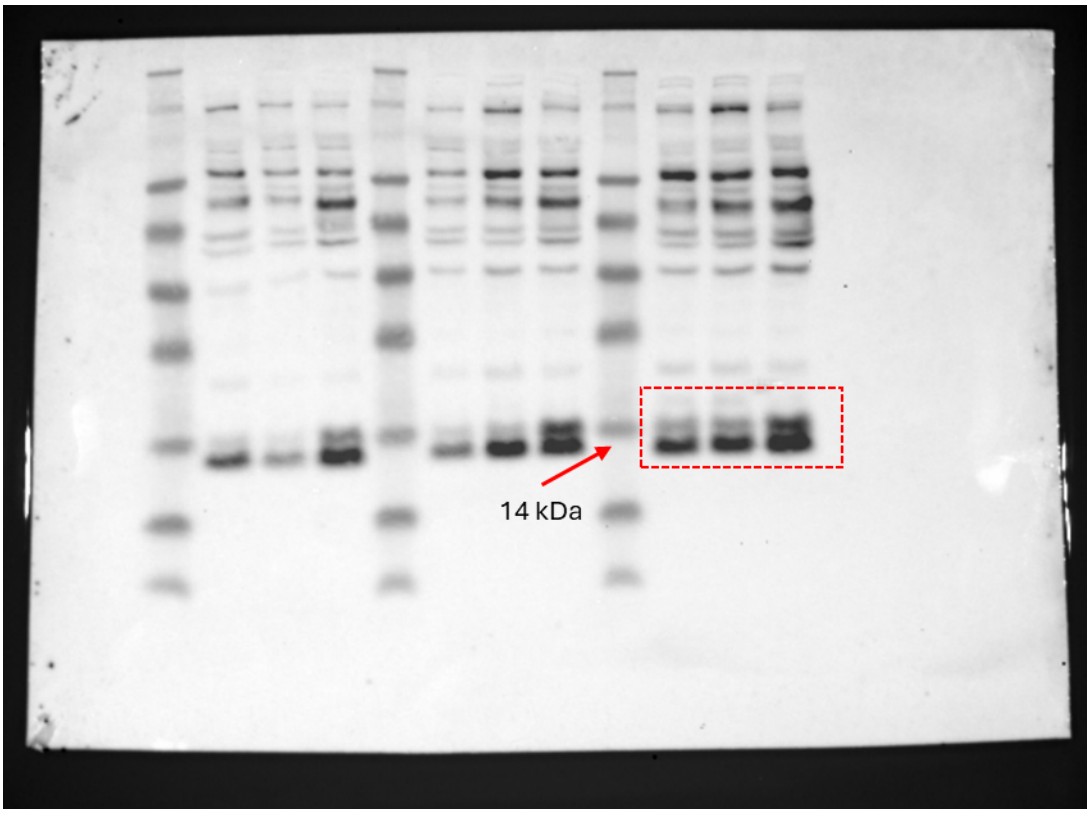

Anti-GAPDH

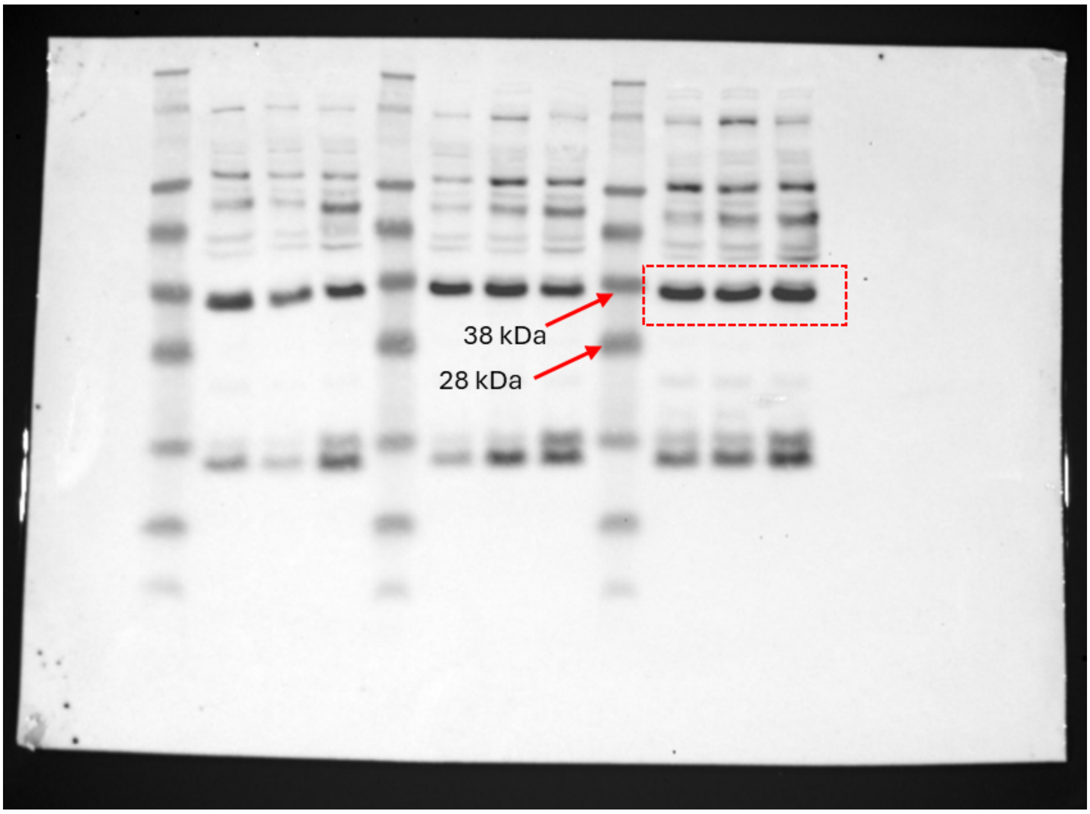

Supplement: Supplementary file 2 — Original Data [file 41419_2026_8984_MOESM2_ESM.pdf]
